# Tumor Evolution in Two Patients with Basal-like Breast Cancer: A Retrospective Genomics Study of Multiple Metastases

**DOI:** 10.1371/journal.pmed.1002174

**Published:** 2016-12-06

**Authors:** Katherine A. Hoadley, Marni B. Siegel, Krishna L. Kanchi, Christopher A. Miller, Li Ding, Wei Zhao, Xiaping He, Joel S. Parker, Michael C. Wendl, Robert S. Fulton, Ryan T. Demeter, Richard K. Wilson, Lisa A. Carey, Charles M. Perou, Elaine R. Mardis

**Affiliations:** 1 Department of Genetics, University of North Carolina at Chapel Hill, Chapel Hill, North Carolina, United States of America; 2 Lineberger Comprehensive Cancer Center, University of North Carolina at Chapel Hill, Chapel Hill, North Carolina, United States of America; 3 McDonnell Genome Institute, Washington University in St. Louis, St. Louis, Missouri, United States of America; 4 Department of Mathematics, Washington University in St. Louis, St. Louis, Missouri, United States of America; 5 Division of Hematology-Oncology, Department of Medicine, School of Medicine, University of North Carolina at Chapel Hill, Chapel Hill, North Carolina, United States of America; 6 Department of Pathology and Laboratory Medicine, School of Medicine, University of North Carolina at Chapel Hill, Chapel Hill, North Carolina, United States of America; University of Texas Southwestern Medical Center at Dallas, UNITED STATES

## Abstract

**Background:**

Metastasis is the main cause of cancer patient deaths and remains a poorly characterized process. It is still unclear when in tumor progression the ability to metastasize arises and whether this ability is inherent to the primary tumor or is acquired well after primary tumor formation. Next-generation sequencing and analytical methods to define clonal heterogeneity provide a means for identifying genetic events and the temporal relationships between these events in the primary and metastatic tumors within an individual.

**Methods and Findings:**

We performed DNA whole genome and mRNA sequencing on two primary tumors, each with either four or five distinct tissue site-specific metastases, from two individuals with triple-negative/basal-like breast cancers. As evidenced by their case histories, each patient had an aggressive disease course with abbreviated survival. In each patient, the overall gene expression signatures, DNA copy number patterns, and somatic mutation patterns were highly similar across each primary tumor and its associated metastases. Almost every mutation found in the primary was found in a metastasis (for the two patients, 52/54 and 75/75). Many of these mutations were found in every tumor (11/54 and 65/75, respectively). In addition, each metastasis had fewer metastatic-specific events and shared at least 50% of its somatic mutation repertoire with the primary tumor, and all samples from each patient grouped together by gene expression clustering analysis. *TP53* was the only mutated gene in common between both patients and was present in every tumor in this study. Strikingly, each metastasis resulted from multiclonal seeding instead of from a single cell of origin, and few of the new mutations, present only in the metastases, were expressed in mRNAs. Because of the clinical differences between these two patients and the small sample size of our study, the generalizability of these findings will need to be further examined in larger cohorts of patients.

**Conclusions:**

Our findings suggest that multiclonal seeding may be common amongst basal-like breast cancers. In these two patients, mutations and DNA copy number changes in the primary tumors appear to have had a biologic impact on metastatic potential, whereas mutations arising in the metastases were much more likely to be passengers.

## Abstract 2

Charles Perou and colleagues report on somatic mutations in primary tumors and metastases from two patients with basal-like breast cancer.

## Author Summary

**Background:**

- In the United States, 40,000 women die of breast cancer each year, thus making it the second leading cause of cancer-related deaths in women.
- Breast cancer mortality is caused by metastasis, the spread of the cancer beyond the breast to distant tissue sites, including the lungs, brain, and liver.
- Triple-negative breast cancer, defined by lack of expression of the estrogen and progesterone receptors and absent amplification of the HER2 gene, and the basal-like molecular subtype defined by RNA gene expression have earlier occurrences of metastasis, worsened survival, and fewer therapeutic options compared to other breast cancer subtypes.

**Why Was This Study Done?:**

- This study was done to gain an understanding of the underlying genetics leading to breast cancer metastasis and when these changes occur temporally.
- Previous reports of the evolution of breast cancer metastasis have reported one to two matched metastasis sites and did not focus on triple-negative breast cancer, the subtype with the greatest clinical need.

**What Did the Researchers Do and Find?:**

- We identified two patients with triple-negative and basal-like breast cancer with available tissue from the primary breast cancer and multiple matched metastases, and performed DNA whole genome sequencing and RNA sequencing on all tumors to identify the genetic landscape of each tumor and define the genomic evolution of metastases from the primary disease.
- We demonstrate that multiclonal seeding from the primary tumor to the metastases can occur, indicating that metastatic cancers can originate from a collection of different subclones that together seed the metastatic site, rather than each metastasis forming from a single cell.
- We also demonstrate that the majority of functional mutations, those expressed and likely to be driving metastasis, are established in the primary tumor rather than being acquired during the spread of disease.

**What Do These Findings Mean?:**

- This study demonstrates examples of multiclonal seeding of metastases from multiple cell populations in the original breast tumor of a patient with basal-like breast cancer.
- The high degree of similarity between the primary tumor and its metastases gives hope that targetable drivers of metastasis are present in the primary tumor and, if effectively treated, could prevent metastasis.
- A larger cohort of matched primaries with multiple sites of metastases per patient is needed to understand the generalizability of these results and possible evolutionary differences of metastasis across the different subtypes of breast cancer.

## Introduction

Breast cancer patients who die from their disease typically succumb to metastatic disease rather than to the primary tumor. Metastasis is a complex process likely involving many potentially distinct mechanistic steps. Biologically similar tumors vary in their ability to seed distant metastatic sites. Indeed, different molecular intrinsic subtypes of breast cancer as determined by the PAM50 subtype classifier vary markedly in their preferred sites for metastasis [1–3]. The luminal subtypes often metastasize to the bone, HER2-enriched tumors to the lung and liver, and basal-like and claudin-low tumors to the brain, lung, and liver [1,2]. The metastatic process is often described as a slow and continuous process of tumor evolution and acquisition of traits such as increased genomic instability, motility, and the epithelial-to-mesenchymal transition. Recent work in renal, prostate, ovarian, and lung cancer has identified significant amounts of intratumor variability in the primary tumor, as well as identifying new driver mutations that arose in metastases [4–8]. In several breast cancer analyses of targeted gene panels, there was considerable concordance of mutations observed between primary tumors and matched metastases [9–12]. This finding, combined with our increasing understanding that a particular intrinsic subtype predicts the future site(s) of metastasis, suggests that in breast cancer at least some of the metastatic potential already exists within the primary tumor [1–3,10]. To examine this further, we studied the genomic relationship between the primary tumors and multiple matched metastases of two patients with triple-negative breast cancer (TNBC), with both cases also of the basal-like breast cancer intrinsic subtype.

A common means of studying intratumor heterogeneity is to sample multiple parts of the same tumor and then perform genetic or genomic assays on these different regions. A more extreme approach to intratumor heterogeneity is to study a primary tumor and its associated metastases to determine the extent to which the metastatic tumor genome was derived from the primary tumor cells as opposed to being an independent tumor [5–7,13,14]. Whether metastases can develop from the primary tumor or require continued evolution and gain of additional mutations in order to metastasize remains unknown in basal-like breast cancers, and addressing this issue may have important implications for therapy. In order to study the genomic evolution of basal-like breast cancer, we performed DNA whole genome and mRNA sequencing on two patients with matched primary tumors and multiple distant metastases.

## Methods

### Patient Consent and Tissue Processing

Tumor tissue was obtained from metastatic breast cancer patients who consented to a rapid autopsy at the University of North Carolina prior to death. Patient consent for the autopsy was obtained in accordance with the UNC Office for Human Research Ethics (OHRE) and criteria established by the US Department of HHS, but this consent procedure was not IRB regulated. There was no prospective analysis plan for this study. For Patient A7, a diagnostic skin punch biopsy of the primary tumor was collected under protocol LCCC 9819 (NCT01000883) as a fresh-frozen sample. For Patient A1’s primary, all metastatic tissues, and normal tissues from both patients, collection was within 6 h of death for all metastatic sites identified prior to death and at time of autopsy. Tissues were frozen in liquid nitrogen, and RNA and DNA were isolated from each tissue using Qiagen RNAeasy and DNAeasy kits, respectively, according to manufacturer protocol (Qiagen, Valencia, California) (S1 Table).

### RNA-Seq

RNA was isolated with RNeasy Mini Kit (Qiagen), and sequencing libraries were prepared with Illumina TruSeq RNA Sample Prep Kit (CAT #RS-122-2001) with the polyA select protocol, except for the A7-Brain, which was first prepared using the Epicentre’s Ribo-Zero rRNA Removal kit (Cat #RZH11042) [15]. RNA-seq was mapped with MapSplice [16] and quantified with RSEM [17]. Upper-quantile normalized counts, log2 transformed, were combined with the Cancer Genome Atlas (TCGA) breast RNA-seq data [18]. Samples were median centered and clustered using the human breast cancer intrinsic gene set list [19], in Cluster 3.0 [20] and visualized with Java TreeView v. 1.1.6r4 [21].

### Illumina Library Construction and Whole Genome Sequencing

A previously described procedure was followed for library construction and sequencing [22]. Briefly, DNA was sheared (Covaris), end repaired (Lucigen), polyadenylated (Lucigen), and ligated to adapters (Illumina) for paired-end data generation. DNA sequencing was performed on the Illumina Genome Analyzer II and generated between 114 and 260 Gbp of sequence data for each tissue studied and haploid coverage ranging from 29.24 to 72.17 (S2 Table).

### Mutation Detection Pipeline

Reads were aligned to human reference build 36 (ftp://ftp.ncbi.nih.gov/genomes/H_sapiens/ARCHIVE/BUILD.36.3/special_requests/assembly_variants/; BWA 0.5.5, http://sourceforge.net/projects/bio-bwa/), merged into a single binary alignment map (BAM) file, with duplicate reads removed using Picard 1.07 (http://broadinstitute.github.io/picard/) by the established pipeline, as previously reported [23]. To determine somatic variants, we utilized samtools [24] followed by SomaticSniper using a somatic score ≥40 and mapping quality ≥40 [25,26]. Additional screening against dbSNP was used to remove probable germline variants [27,28]. Indels were identified with Pindel [29] and GATK [30]. All variants were further annotated as previously described [22,27] using VarScan2 [31] (parameters: --*min-coverage* = 30,* --;min-var-freq* = 0.08,* --normal-purity* = 1,* --p-value* = 0.10,* --somatic-p-value* = 0.001,* --validation* = 1) to classify mutations as reference, germline, somatic, or resulting from loss of heterozygosity (LOH). A Bayesian classifier was applied to retain the somatic variants with a binomial log-likelihood of at least 3 (parameters: --*llr-cutoff* = 3, --*tumor-purity* = 0.95). False positives, as determined by strand specificity, consistent positions near the ends of reads, and poorly mapped qualities were removed.

Mutations were assigned to four tiers: (1) coding, (2) conserved or regulatory, (3) unique noncoding, and (4) repetitive noncoding regions.

### Structural Variant Detection

Structural variants (SVs) were called with BreakDancer [32] and filtered using TIGRA_SV [33]. Somatic copy number alterations were detected using CopyCat v1.6.9 (https://github.com/chrisamiller/copycat), with 10,000 bp windows and default parameters.

### Experimental Validation of Mutations

#### SNP arrays

Genotypes from Illumina Human OmniExpress BeadChip SNP arrays were used to compare and confirm the heterozygous SNPs detected in the analyzed WGS data.

#### Small (1–2 bp) indels

Putative indels of 1–2 bp were converted to BED format and provided as target intervals for the GATK IndelRealigner [30,34]. The primary, metastases, and matched normal breast tissue for each patient were then realigned to these BED files independently. To validate the original predictions, we developed a matching algorithm that attempts to match Varscan validation calls with the original indel predictions, as described [23]. All validated somatic indels were then manually reviewed using Integrative Genomics Viewer (IGV) [35].

#### Medium (3–100 bp) indels

Indels of 3–100 bp were assembled using TIGRA [33] and validated as previously described [23]. Variants that passed the strict validation were manually reviewed.

#### Solid phase capture

Custom sequence capture validation was performed with Roche NimbleGen arrays for 97.3% of the Tier 1–3 somatic alterations and 68.6% of the SVs. Whole genome amplified DNA was prepared for Illumina sequencing according to the manufacturer’s protocol (Illumina, San Diego, California). DNA was fragmented with the Covaris S2 DNA Sonicator (Covaris, Woburn, Massachusetts), adapter-ligated, SPRI-bead cleaned, and PCR amplified. One μg of the 300–500 bp fragment library was hybridized to the NimbleGen HD2 probe set according to the manufacturer’s protocol (Nimblegen, Madison, Wisconsin). Following hybridization, the library was PCR amplified for 16 cycles and quantified with the KAPA SYBR FAST qPCR Kit (KAPA Biosystems, Woburn, Massachusetts) and diluted such that 180,000 clusters were sequenced per lane of the Illumina GAIIx.

Reads were mapped to the NCBI Build 36 reference WUGSC Variant, which is a subset of the NCBI36 sequences from Ensembl Release 46 (full assembly at ftp://ftp.ncbi.nih.gov/genomes/H_sapiens/ARCHIVE/BUILD.36.3/special_requests/assembly_variants/).

The validation sequence was aligned with BWA v0.5.9, and duplicate reads were marked using Picard (v1.29). Updated versions of BWA and Picard were used for increased alignment speed and variant detection efficiency. The RefCov package was used to evaluate the coverage of target sequences (http://gmt.genome.wustl.edu/packages/refcov/).

#### Validation of SVs

Capture validation reads and mates were mapped to both the assembled SV contigs and the reference with CrossMatch (version 1.080721). The threshold for an acceptable alignment is ≤1 mismatch at either end, ≤1% substitutions, 1% indels, and a CrossMatch score ≥ 50. An SV-supporting read is required to span the breakpoint on the SV contig, align to 10 bases flanking on each side of the breakpoint, and have no alignment to the reference above the minimum alignment criteria. The somatic status of each SV was determined using Fisher’s exact test between the matched tumor and normal sample. All validated calls were manually reviewed.

### RNA Validation of Mutations

UNCeqR [36] was run on validation mode for all samples. In validation mode, the algorithm accepts as input a set of predetermined mutations, such as a list of mutations generated from WGS/WES, and then looks within the RNA-seq data for expression evidence of the variants. Tier 1 mutations were input into UNCeqR along with the RNA-seq BAM files aligned with MapSplice [16]. UNCeqR then calculated the number of reads of the reference and variant alleles at each position interrogated. Mutations with less than 5 reads were considered as 0. RNA variant allele fraction (VAF) was calculated as variant allele reads/total reads.

### Clonality Analyses

The clonal structure of each tumor was inferred with SciClone (version 1.0.7) [37], with parameters minDepth = 75, copyNumberMargins = 0.25, and maximumClusters = 20. Single nucleotide variants (SNVs) in copy number altered regions or with evidence of complete or partial LOH were reviewed and excluded. Phylogeny was inferred using the clonevol R package (https://github.com/hdng/clonevol) with default parameters.

## Results

### Case Histories

**Fig 1:**
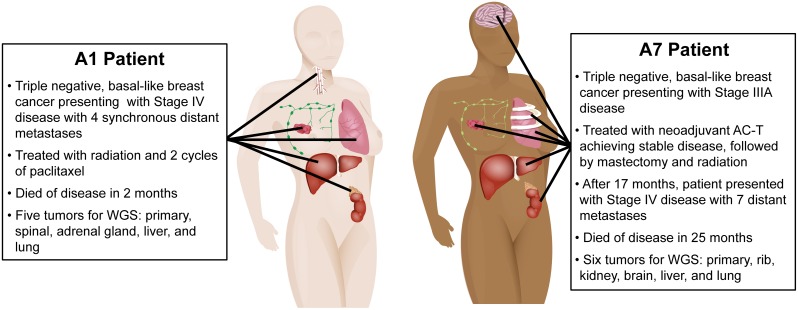
Clinical history and distribution of metastases from patients A1 and A7, who both had clinically triple-negative and basal-like breast cancer.

Patient A1 was a 65-y-old white woman who presented with stage IV TNBC and synchronous metastases to the bone of the vertebral column (spinal), lung, adrenal gland, liver, and lymph nodes. She was treated with radiation therapy to the breast, whole brain, and C3/T2 of the spine, had one cycle of palliative paclitaxel without response, and died of disease 2-mo postdiagnosis. Patient A7 was a 60-y-old African-American woman diagnosed with a 5-cm stage IIIA TNBC. A pretreatment primary tumor biopsy was collected as a part of an existing tissue collection protocol (LCCC 9819, NCT01000883), and she subsequently received neoadjuvant doxorubicin plus cyclophosphamide followed by paclitaxel. She underwent mastectomy with T2N2 residual disease, followed by adjuvant radiation therapy to the chest wall (SCV fossa and axillary nodes). Patient A7 remained without evidence of disease recurrence for 17 mo before presenting with metastases to the brain, kidney, liver, lung, and ribs. She received single-agent capecitabine for 4 mo, with an initial minimal response and then progression both systemically and in the central nervous system (CNS), followed by a single cycle of carboplatin that was discontinued because of poor tolerability and evidence of rapid progression. Patient A7 died of disease 8 mo after her metastatic progression. For both patients, fresh frozen tissue was collected at autopsy from primary tumor, distant metastases, and adjacent normal (nonmalignant breast) tissue, except for the primary tumor specimen that was obtained before neoadjuvant treatment was initiated in patient A7 (Fig 1).

### Whole Genome Sequencing Coverage and Mutation

For the matched normal tissues, primary tumor (pretreatment biopsy for A7), and distant metastases from patients A1 and A7, we performed DNA whole genome paired-end sequencing. For A7, we derived 138.38, 118.76, 260.93, 128.69, 204.34, 201.66, and 156.82 Gbp of sequencing data from normal tissue, primary tumor, liver, lung, rib, kidney, and brain metastases, respectively, with corresponding haploid coverages ranging from 33.17X to 70.19X (S2 Table). For A1, we generated 265.53, 134.07, 115.85, 210.45, 114.31, and 131.03 Gbp of data from normal tissue, primary tumor, liver, lung, adrenal, and spinal cord metastases, respectively, with haploid coverage ranging from 30X to 72.16X (S2 Table).

Candidate somatic changes were predicted using multiple algorithms. Confirmatory testing of heterozygous mutations with genotype arrays confirmed biallelic detection of 80.47% to 89.63% in all samples (S2 Table). Candidate mutations were further validated with capture probes corresponding to all putative somatic SNVs and small insertions/deletions (indels) that overlap with coding exons, splice sites, and RNA genes (Tier 1), a number of high-confidence SNVs and indels in noncoding conserved or regulatory regions (Tier 2), and nonrepetitive regions of the human genome (Tier 3). In addition, we included predicted somatic SVs for validation. We obtained 40X haploid reference coverage for 87.48% to 94.02% of the targeted sites (S3 Table). For A1, 73 Tier 1 point mutations, 1 Tier 1 indel, and 53 somatic SVs were confirmed across the primary tumor and metastases (S4–S6 Tables). For A7, there were 150 Tier 1 point mutations, 47 indels, and 40 SVs confirmed in the primary tumor and five metastatic samples (S7–S9 Tables).

### Genomic Relatedness of Primary Tumors and Metastases

#### Common gene expression patterns throughout metastasis

**Fig 2:**
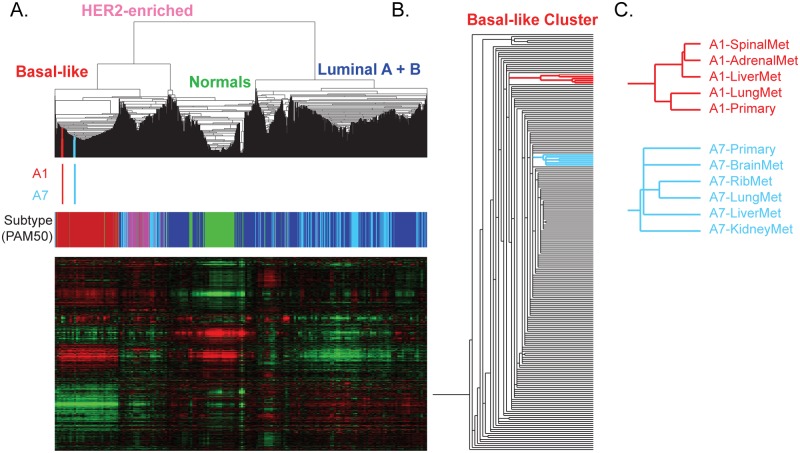
Molecular relatedness of matched primary and metastases. (A) Hierarchical clustering of patient A1 and A7’s tumors with 1,100 TCGA Primary samples and 98 normal breast samples analyzed using a breast cancer intrinsic gene list. The color bars under the dendrogram indicate (i) where A1 (red) and A7 (blue) specimens are clustered and (ii) the PAM50 subtype of each sample (basal-like, red; HER2-enriched, pink; luminal A, dark blue; luminal B, light blue; and normal-like, green). (B) The position of A1 (red) and the position of A7 (blue) within the basal-like cluster are highlighted. (C) The relationship of the primary and metastases for each patient based upon gene expression patterns.

In order to study the degree of relatedness between a primary tumor and its metastases, we performed mRNA-seq gene expression analyses followed by hierarchical clustering analysis using a breast cancer “intrinsic” gene list [19] including data from the 11 specimens studied here and 1,100 breast tumors from the TCGA Project [18]. Regardless of physical or temporal distance between the primary and its metastases, all tumors from these two patients clustered tightly together by patient (Fig 2). By gene expression analysis using the PAM50 intrinsic breast cancer subtype predictor [19], the primary tumors and metastases all maintained a basal-like subtype phenotype and clustered with the basal-like samples from TCGA (Fig 2B); previous research has demonstrated a high correlation among primaries and matched metastases by microarray gene expression [1,38]. In patient A1, in whom the primary tumor and distant metastases were found synchronously and who had limited exposure to chemotherapy and radiation prior to death, the gene expression hierarchical cluster node correlation for the primary and the four metastases was 0.77 (Fig 2C). In patient A7, who received neoadjuvant chemotherapy and radiation and had a 17-mo interval separating the discovery of the primary tumor and distant metastases, the node correlation for the six samples was 0.79 (Fig 2C). This demonstrates that subtype was maintained throughout metastasis in these two patients and that, as we and others have shown [1,38], distant metastases are typically much more similar to their original primary than they are to other primary tumors or metastases from other patients.

#### Functional mutations are maintained and enriched during metastasis

We next studied DNA-based data from each primary tumor and its multiple distant metastases. In patient A1, 54 genes were mutated with a VAF greater than 0.5% in the primary tumor (13 nonsilent mutations were in the Catalogue of Somatic Mutations in Cancer [COSMIC] [39]) (S4 Table). Almost every Tier 1 mutation present in the A1 primary tumor was identified in one or more of the metastases (52/54), and in many cases the VAF was enriched in the metastasis (median: 5-fold enrichment, average: 8.8-fold, range: 1- to 38-fold; S4 Table, S1 Fig). Eleven mutated genes were shared among the primary and all matched metastases: *TARBP1*, *FCRL1*, *XIRP1*, *TRMT1*, *PANX3*, *MYSM1*, *PHLDB3*, *TBC1D25*, *LOC284288*, *MDS2*, and *TP53*. The adrenal metastasis and spinal metastasis contained the most unique SNVs, with seven and nine, respectively. The liver metastasis and lung metastasis did not have any private mutations at a VAF > 1%, although the lung metastasis did share two mutations with the adrenal metastasis that were not observed in the primary.

In patient A7, 75 Tier 1 genes were mutated with a VAF ≥ 0.4% in the primary tumor (14 of these nonsilent mutations were in COSMIC) (S7 Table, S1 Fig). The VAF in all of the metastases had a median enrichment of 1.4-fold, closer to the primary tumor than in patient A1. All of the mutations identified in the primary tumor were detected in at least one metastasis, and 65 mutations, including mutations in *RUNX1T1*, *ADGRB2*, *KMT2C*, *RP1*, *TP53*, and *AKT3*, were shared across the primary and all matched metastases. There were 75 mutations identified in one or more of the metastases that were not observed in the primary tumor (8 of these nonsilent mutations also were in COSMIC). The majority of these metastasis-specific mutations (54/75) were present in two or more metastases. Of the 21 mutations private to a single metastasis, the liver and kidney metastases had the most, with 7 and 8 private mutations, respectively. The rib metastasis contained no unique mutations.

#### *TP53* as a common driver of metastasis

*TP53* alterations are frequently observed in basal-like breast cancers [40]. *TP53* was the only shared somatic mutated gene between the two patients and was present in every tumor specimen sequenced. Close examination of patient A1 data identified an 11 bp deletion in *TP53* that was common to all samples (S2 Fig). In patient A7, the *TP53* missense mutation H168R had a greater than 68% VAF in all tumors except the brain metastasis (31%). While this exact mutation was not observed in the TCGA breast cohort, a missense mutation was identified at the same position in one case (H168P) [41,42], supporting the likelihood that alteration of *TP53* is a founding event critical for the development of basal-like breast cancer [43] and subsequent metastasis.

#### Mutations established early tend to be expressed and enriched in metastasis

**Fig 3:**
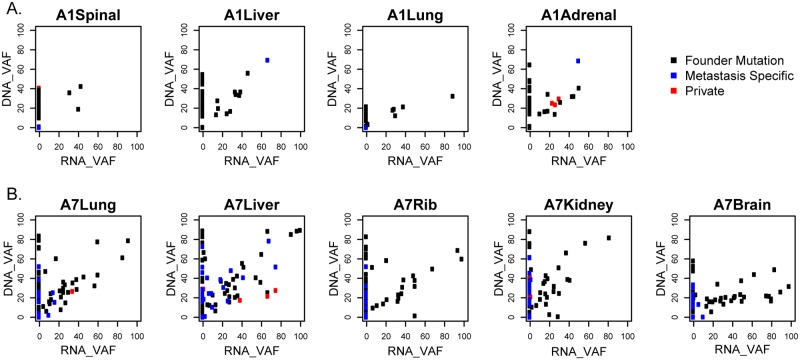
Gene expression of variant alleles. Variant allele fractions (VAFs) of each point mutation were determined from mRNA-sequencing data and compared to those from combined whole genome sequencing (WGS) and validation sequencing data. Gene variants shared in the primary and metastases (shared mutations, black), metastases but not primaries (metastases specific, blue), or only in one metastasis (private, red) in patients A1 (A) and A7 (B) are shown.

We examined the mRNA expression data for evidence of expression of the somatic point mutations in primary tumors and metastases. Interestingly, mutations shared between the primary and metastatic tumors were more likely to be expressed (Fig 3, black dots) and were expressed at higher levels than mutations unique to metastasis (Fig 3, blue dots). In patient A1, 21/52 (40%) of the mutations established in the primary were expressed in the metastases (Fig 3A, black dots). In patient A7, 47/75 (63%) of the mutations established in the primary were expressed both in the primary and in the metastases (Fig 3B, black dots).

Fewer mutations were detected only in the metastases, and those mutated transcripts had lower RNA expression than mutations shared with the primary (Fig 3, blue dots). In patient A1, 2 of the 3 mutations shared among more than one metastasis but not in the primary tumor were expressed (Fig 3A, blue dots), while only 4 of the 18 private mutations (detected only in individual metastases) were expressed (S4 Table, Fig 3A, red dots). In patient A7, 23/54 (43%) of the mutations that were shared across the metastases but not with the primary tumor were expressed, and 8/21 (38%) of the private mutations were expressed (S7 Table, Fig 3B).

Interestingly, many of the expressed metastasis-specific mutations occur in genes that are involved in DNA damage responses, RNA processing, and degradation of the extracellular matrix (ECM). In patient A1, metastasis-specific mutations included *FANCF* and *SMC6* (DNA double-stranded break repair), *DDX6* (promotes mRNA degradation), and *HYAL3* (degrades hyaluronan in the ECM) [44]. In patient A7, *AQR*, *DOCK6*, and *HLTF* were shared across metastases and expressed. Metastasis-specific mutations in patient A7 included *CASC3* (the core of the exon junction complex), *TIMP3* (degrades ECM), and *LAMA5* (part of the ECM) [44]. These could represent convergent evolutionary paths to the resistance of DNA damaging agents and promotion of cell mobility and survival.

#### Structural variations tend to be established early in metastasis

To further explore the development of larger genomic alterations during metastasis, Circos plots were generated to illustrate the combined Tier 1 somatic mutations, DNA copy number alterations, and SVs for each sequenced tumor (patient A1: S4–S6 Tables, S3 Fig; patient A7: S7–S9 Tables, S4 Fig). These illustrate that, overall, SVs were mostly established in the primary tumor and maintained through the different metastatic processes.

In patient A1, all 8 of the SVs in the primary tumor were shared with the metastases (S3 Fig), including one that was specifically shared with the adrenal and liver metastases (S6 Table). The metastases had few additional interchromosomal SVs, and these were shared, except in the spinal metastasis. Interestingly, the spinal metastasis evolved to have many more rearrangements between chromosomes 2 and either 3, 8, 12, or 16.

In patient A7, the brain and kidney metastases shared most interchromosomal SVs with the primary (S4 Fig, S9 Table). The rib and liver metastases had three private SV alterations each (of a total of six and eight alterations, respectively), while the lung metastasis showed many more private interchromosomal SVs than the other metastatic samples.

#### FBXW7-INPP4B fusion in patient A7

To confirm SVs, we created a modified genome that represented the possible new alignments in RNA space. Realigning A7 data to this map demonstrated expression of an *FBXW7-RNF150* fusion gene observed in all A7 samples, indicating early fusion of this gene in the development of this patient’s breast cancer (S5 Fig). Interestingly, deletion of the last ten exons of *FBXW7* was previously reported as a founding event in a basal-like breast cancer [45]. The 5′ end of the fusion in patient A7 began at exon 3 or 4 of *FBXW7*, which likely inactivated *FBXW7*. The 3′ end of the fusion occurred just before *RNF150*, resulting in deletion of *INPP4B*. There was decreased RNA expression of *INPP4B* in this patient, further supporting the deletion of *INPP4B* by the *FBXW7-RNF150* fusion gene event. *INPP4B* has important implications in breast cancer that include DNA repair defects [46], increased genomic instability [47], and inhibition of the PI3K pathway [48].

### Multiclonal Evolution of Metastasis in Two Patients with TNBC

**Fig 4:**
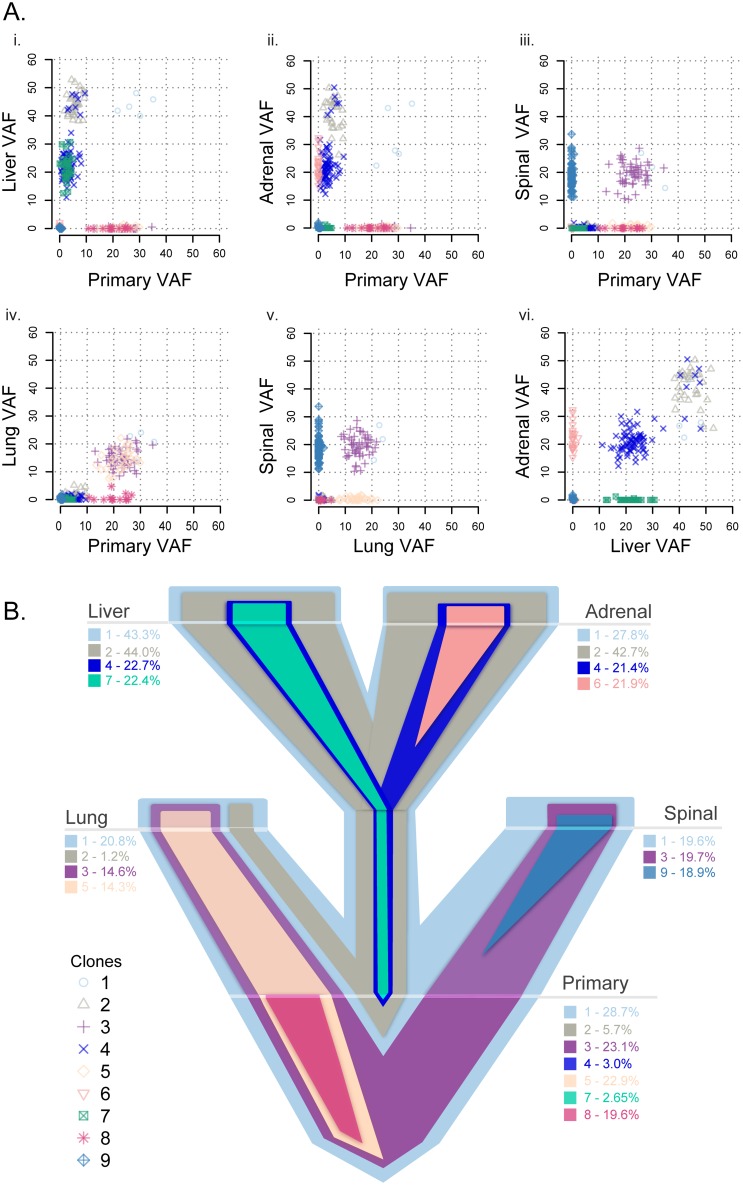
Clonality analysis of each tumor from patient A1. VAFs among the primary and matched metastases in patient A1 (A) and a representative evolutionary tree (B) colored by subclone based on the clonality plots in panel A, with the width of the branch indicating the approximate percentage of that clone within the tumor. Clone 1 is established in the primary tumor and seeded all distant metastases. Clones 2 and 4 from the primary tumor seeded the liver and the adrenal gland, with clone 7 concurrently seeding the liver from the primary tumor. Clones 3 and 5 from the primary tumor seeded the lung, with clone 3 also seeding the spine. Private clones include clone 6, specific to the adrenal metastasis; clone 8, specific to the primary tumor; and clone 9, specific to the spinal metastasis.

**Fig 5:**
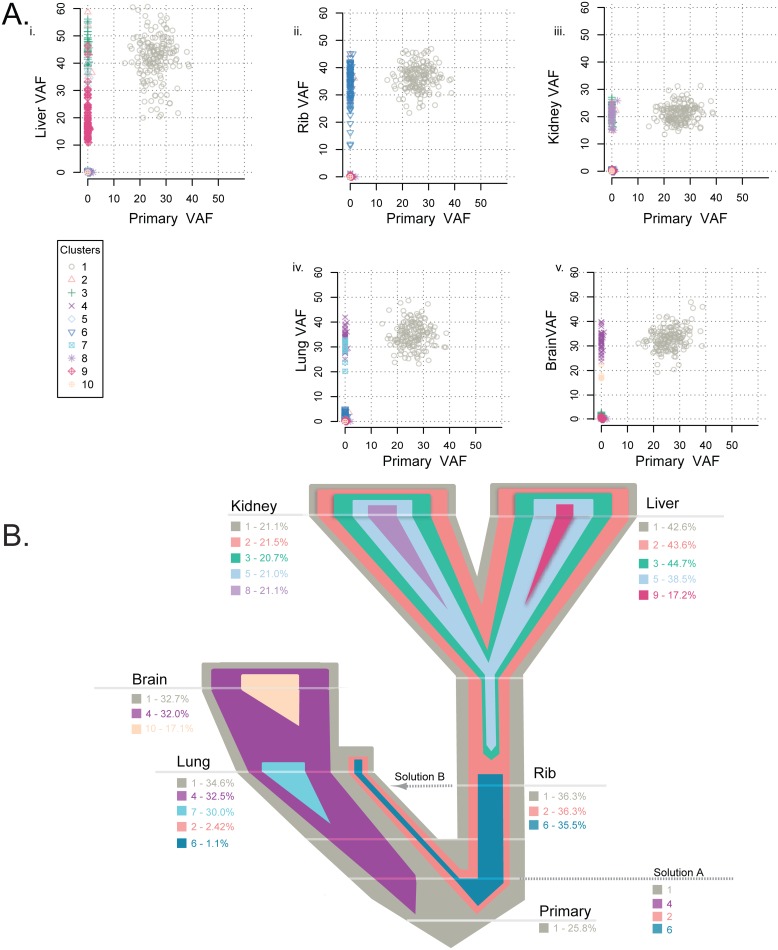
Clonality analysis of each tumor from patient A7. Clonality shared among the primary tumor and matched metastases in patient A7 (A) and the representative evolutionary tree (B) colored by subclone identity based on the clonality plots in panel A, with the width representative of the percentage of the clone within that tumor. Clone 1 was established in the primary tumor and maintained through metastatic spread in every tumor. Clone 2 was present in the liver, kidney, and rib and at a low frequency in the lung, while clones 3 and 5 were shared by the liver and kidney metastases. Clone 6 was present in the rib and a low frequency in the lung metastases. Brain and lung metastases shared clone 4. Four metastases had a private clone not shared with any other tumor: clone 7 specific to the lung, clone 8 specific to the kidney, clone 9 specific to the liver, and clone 10 private to the brain.

To understand the Darwinian evolution occurring in the primary tumor and throughout metastasis [49], we established the subclonal relationships and phylogenetic trees for patient A1 (Fig 4, S6–S8 Figs, S10 Table) and patient A7 (Fig 5, S9 and S10 Figs, S11 Table).

Subclonality analysis using Sciclone of the A1 patient samples demonstrates that the primary tumor predominantly contained clones 1, 3, 5, and 8, with very low allele fractions of minor clones 2, 4, and 7 (S6 Fig, Fig 4A). Clone 1, established in the primary tumor, seeded all other metastases. Of the other major clones in the primary, clones 3 and 5 seeded the lung metastasis, while clone 3 additionally seeded the spinal metastasis. This metastasis then continued to evolve, developing private clone 9 (Fig 4A, S6 Fig). These clones (3 and 5) were mutually exclusive with minor clone 2, which was found in the primary tumor, lung, liver, and adrenal metastases (S7 Fig). Two of the minor clones in the primary tumor (clones 2 and 4) became the dominant clones in the liver and adrenal metastases, with additional private subclonal evolution in the adrenal metastasis (clone 6). Interestingly, clone 7 was established in the primary tumor and also metastasized to the liver, but not to the adrenal, metastasis (Fig 4B). Using ClonEvol, there were two potential models for clone 7 development that we were not able to fully resolve; either it evolved (1) from clone 4 (S7A Fig) or (2) independently from clone 2 (S7B and S8 Figs). This result demonstrates that the multiclonal metastatic potential residing in the primary tumor is maintained through metastasis.

Importantly, patient A1 presented at stage IV and only received two doses of radiation and one cycle of single-agent taxane before death. Thus, her primary-to-metastatic disease likely is representative of the natural course of basal-like breast cancer rather than representing selection from the evolutionary pressure imposed by therapy.

In patient A7, the subclonal structure was determined by SciClone (S9 Fig, S11 Table), and a single model of evolution was suggested by ClonEvol (S10 Fig). The primary tumor consisted of one main clone (Fig 5A), seeding all other sites of metastasis at the highest VAF observed. The main clone then diverged to two lineages, giving rise to clone 2 predominantly in the liver, kidney, and rib and clone 4 predominantly in the lung and brain (Fig 5B). Clone 4 is present in the lung and brain metastases at an almost equivalent VAF to the founding clone 1. Clones 2 and 6 in the rib are also present at an almost equivalent VAF to clone 1; clones 2 and 6 are seen at a low VAF in the lung. These clonal data paint a complex picture with two possible explanations: either the split of clone 1 into clones 2 and 6 and clone 4 occurred prior to metastatic spread (Solution A, Fig 5B) or these clones cross seeded from the rib metastasis to the lung metastasis (Solution B, Fig 5B). Clone 2 further evolved to clones 3 and 5 in the liver and kidney metastases. We favor the first hypothesis, namely that clone 2 in the rib, liver, and kidney metastases is at a VAF equivalent to the founding clone, indicating that the evolution of this clone occurred before metastatic seeding. All metastases aside from the rib metastasis also contained private subclones.

## Discussion

Whole genome sequencing and mRNA sequencing of two TNBC/basal-like breast cancer patients with primary tumors and multiple matched metastases demonstrated significant genetic similarity between the primary breast cancers and their matched metastases. Patient A1 demonstrated significant intratumoral heterogeneity established in the primary tumor and multiclonal seeding of metastasis. Interestingly, patient A7 possibly contained a more homogenous primary breast cancer that then led to diverse, heterogeneous metastases. Even though there is continued evolution, the acquisition of mutations private to a single metastasis likely had limited impact on the metastatic potential, as these mutations were rarely expressed or were expressed at low levels. In contrast to earlier findings in renal cell carcinoma of monoclonal metastasis seeding [4], basal-like breast cancer metastases can be the result of multiclonal seeding of cells established in the primary. The results presented here are inconsistent with a single cell of a primary breast cancer seeding a distant metastasis [50]. Herein, we describe an example of multiple subclones that resided within a primary tumor followed by multiclonal seeding of all distant metastases as well as a common disruption of *TP53*.

In both patients, relatively few mutations occurred once the tumor cells left the primary site, and of those that did alter protein coding sequences, the mutations were not highly expressed at the RNA level in general. The high correlation of gene expression among primaries and matched metastases illustrates that subtype is typically maintained throughout metastasis [1], and that specific intrinsic subtypes have an inherent tendency to metastasize to specific organs [1,3]. Taken together, these results suggest that the metastatic potential was present within the primary tumor of these two basal-like breast cancer patients. Here, we uncover a genetic explanation for the close correlation of gene expression in metastases and matched primaries—namely that, in the two cases examined, the samples from a given individual were much more genetically similar than they were dissimilar, both on the DNA and RNA levels.

While the majority of genetic alterations present in metastases were shared with the matched primary cancer in these two patients examined, we also identified a significant amount of intratumoral heterogeneity, evident because multiple subclones were detected within each metastasis. Patient A1 demonstrates that more than one subclone from the primary seeded each metastasis, and the intratumoral heterogeneity in the primary tumor setting was mostly reflected in each metastasis. In patient A7, the lung metastasis exhibited diverse intratumoral heterogeneity, with two small subclones (2 and 6) found at high frequency in three of the other metastases. There are two possible explanations for the complex clonal patterns seen in patient A7: either the two dominant clones (clones 2 and 4) were established in the primary and were not sampled in the piece of the primary tumor that was actually sequenced, or clones 2 and 6 in the rib cross seeded into the lung metastasis. While one metastasis seeding another metastasis has been previously demonstrated in prostate cancer [7], we also recognize that the A7 primary breast cancer likely had spatial heterogeneity that was not fully captured by our sequencing [51]. In fact, the A7 primary breast cancer piece sequenced was a skin punch biopsy taken from a 5 cm primary breast cancer, rather than a tumor resection. Hence, samples from multiple portions of this tumor were not sequenced. Of the two possibilities, the most parsimonious explanation for the observations relevant to patient A7 is that multiclonal seeding of the metastases did occur and that our limited sample did not permit detection of clones 2 and 6. Hence, only subsequent deep sequencing of additional portions of the A7 tumor would resolve the issue of monoclonal versus multiclonal seeding from the primary. Unfortunately, no additional specimens exist for this patient. Regardless of this, in patient A7 multiple multiclonal seeding events were discovered, such as the rib metastasis seeding the kidney and liver.

The genetic heterogeneity in both of the primary tumors and the resulting metastases may explain why many metastatic TNBC patients fail to have a durable treatment response and instead progress within a few years [52]. In particular, heterogeneity provides for a wealth of individual genotypes, thus yielding a genetic diversity from which chemotherapy resistance may arise. Treatment has been shown to select for therapy-resistant clones in primary breast cancer [53–55], and therapy can select for subclones in the metastatic setting.

While our studies provided evidence of multiclonal seeding of metastasis in these two patients, both with basal-like breast cancer, our results may or may not apply to a larger cohort of patients with basal-like breast cancers, to other subtypes of breast cancers, or to other cancer types. Even within the poor-prognosis basal-like subtype, patients often receive many more lines of therapy and have more favorable responses to their therapies for a longer duration than the two patients presented here. Furthermore, patients with luminal and HER2-enriched breast cancer have comparatively more opportunities to benefit from targeted therapies such as tamoxifen, aromatase inhibitors, and/or HER2 agonists such as trastuzumab, lapatinib, or pertuzumab. Since neither patient A1 nor A7 was treated with targeted therapies, there were different selective pressures in the metastatic setting compared to current standard of care for ER+ and HER2+ patients.

The basal-like subtype is a highly aggressive cancer that often metastasizes to the lung and brain within 5 y of diagnosis. This is in contrast to luminal A breast cancers, which are typically more indolent, are less likely to progress to stage IV, and typically metastasize first to the bone [56]. The difference in these patterns of relapse and the timing with which they occur suggest fundamental differences in disease progression between the subtypes [57] within the context of drastically different treatment strategies. Continued analyses of larger datasets representing each of the subtypes and patients with varying clinical histories will be necessary to identify consistently altered genes to define early versus late drivers, metastasis-site specific alterations, and differences among the mechanisms of metastasis across various subtypes of breast cancer.

## Supporting Information

S1 Appendix Supplementary Materials and Methods. In-depth description of the capture array design, paired-end library preparation, and solid phase capture. Further description of bioinformatic methods for clonality and determining significantly mutated genes.
(DOCX)

S1 Fig Heat map of the DNA variant allele frequency of Tier 1 mutations in patients A1 and A7. The vertical bar to the left of each heat map designates genes shared with the primary and metastases (black), genes mutated in metastases but not in the primary (blue), and genes private to a single individual metastasis (red) in (A) Patient A1 and (B) Patient A7.
(PDF)

S2 Fig *TP53* Deletion in A1. Genome view of the 11 bp deletion of *TP53* in Patient A1 at chr17:7,579,474 to chr17:7,579,485, present in the primary tumor and all of the metastases.
(PDF)

S3 Fig DNA alterations of matched primary and metastases of patient A7. (A–F): Circos plot displays mutations, copy number, and structural rearrangements in the (A) primary, (B) spinal, (C) lung, (D) liver, and (E) adrenal metastases. Translocations with significant read coverage include shared (green) and private (red) interchromosomal and shared (purple) and private (blue) intrachromosomal translocations.
(PDF)

S4 Fig Circos plots of matched primary and metastases of patient A7. (A–F): Circos plots displaying mutations, copy number landscape, and structural rearrangements (order starting from outside) in the (A) primary, (B) rib, (C) kidney, (D) liver, (E) brain, and (F) lung metastases. Translocations with significant read coverage include shared (green) and private (red) interchromosomal and shared (purple) and private (blue) intrachromosomal translocations.
(PDF)

S5 Fig FBXW7 fusion. Representative illustration of FBXW7 fusion and INPP4B deletion in all tumors from A7.
(PDF)

S6 Fig SciClone analysis of A1. SciClone analysis of variant allele frequencies in copy number neutral regions of each tumor using Bayesian beta mixture modeling and multi dimensional clustering of tumors from patient A1. Multiple clones are shared in the primary and metastases, with Clone 1 in the primary and all matched metastases; Clone 2: primary, adrenal, and liver; Clone 3: primary, adrenal, and liver; Clone 4: primary, lung, and spine; Clone 5: primary, adrenal, and liver; Clone 6: primary and lung; Clone 7: adrenal; Clone 8: primary and liver; Clone 9: primary; and Clone 10: spinal.
(PDF)

S7 Fig ClonEvol analysis of A1. ClonEvol demonstrates that Clones 1 and 2 are founding clones that seed the distant metastases at different percentages. Clone 2 and Clone 3 are exclusive of one another, leading to separate lineages. The proportion of each clone for each tumor is demonstrated by the width of the nested shapes. Two possible models are presented.
(PDF)

S8 Fig Representative evolutionary tree of an alternative model of A1. ClonEvol predicted two possible evolutionary lineages of clones in patient A1. The first model is in Fig 5B. The alternative model demonstrating that Clone 7 is independent of Clone 4 is presented.
(PDF)

S9 Fig SciClone analysis of variant allele frequencies of patient A7. SciClone analysis of only copy number neutral regions demonstrates multiclonal seeding of metastases. The Lung metastasis contains both branches of the clonal tree, predominantly containing Clone 4 but with a small fraction of Clone 2. In Contrast, the rib metastasis contains predominantly Clone 2 with a small minority of Clone 3. Private clones are seen in all metastases.
(PDF)

S10 Fig Clonevol analysis of A7. ClonEvol of the copy number neutral mutations from SciClone analysis demonstrates one founding clone leading to a branched pattern of Clones 2 and 4. Private clones are present in all metastases.
(PDF)

S1 Table Sample descriptions. Sample information for RNA and DNA sequencing runs, including the name of matching fastQ files that have been uploaded to the Database of Genotypes and Phenotypes (dbGAP) (accession number phs000676.v1.p1) as well as the clinical information of Patients A1 and A7.
(XLSX)

S2 Table Whole genome coverage. Includes coverage of input base pairs (Gb), haploid coverage, and concordance with heterozygous SNPs.
(XLS)

S3 Table Capture validation. 40X, 30X, and 20X capture validation of whole genome sequencing for all samples in patients A1 and A7.
(XLS)

S4 Table Mutational landscape of A1. Variant allele frequencies for variants in both DNA and RNA. RNA samples with less than five reads were considered as having a VAF of 0 for analysis purposes. The RNA VAF is the percent of reads at each loci with the variant in the RNA sequencing identified from the DNA whole genome sequencing.
(XLSX)

S5 Table DNA copy number alterations of A1. (XLS)

S6 Table Intrachromosomal and interchromosomal translocations of tumors from patient A1. (XLS)

S7 Table Mutation landscape of A7. Variant allele frequencies for the mutation in both DNA and RNA for each gene. RNA samples with less than five reads were considered as having a VAF of 0 for analysis purposes. The RNA VAF is the percent of reads at each loci with the variant in the RNA sequencing identified from the DNA whole genome sequencing.
(XLSX)

S8 Table DNA copy number alterations of A7. (XLS)

S9 Table Intrachromosomal and interchromosomal translocations of tumors from patient A7. (XLSX)

S10 Table Clonality analysis of A1. The table includes gene names, position, read depth, DNA mutation, and cluster assignment for each mutation in copy number neural positions used for clonality analysis.
(TXT)

S11 Table Clonality analysis of A7. The table includes gene names, position, read depth, DNA mutation, and cluster assignment for each mutation in copy number neutral positions used for clonality analysis.
(TXT)

## Abbreviations

CNS: central nervous system
COSMIC: Catalogue of Somatic Mutations in Cancer
dbGAP: Database of Genotypes and Phenotypes
ECM: extracellular matrix
SNV: single nucleotide variant
SV: structural variant
TCGA: the Cancer Genome Atlas
TNBC: triple-negative breast cancer
VAF: variant allele fraction
WGS: whole genome sequencing
