# Correction: Tumor Evolution in Two Patients with Basal-like Breast Cancer: A Retrospective Genomics Study of Multiple Metastases

**DOI:** 10.1371/journal.pmed.1002222

**Published:** 2017-01-09

**Authors:** Katherine A. Hoadley, Marni B. Siegel, Krishna L. Kanchi, Christopher A. Miller, Li Ding, Wei Zhao, Xiaping He, Joel S. Parker, Michael C. Wendl, Robert S. Fulton, Ryan T. Demeter, Richard K. Wilson, Lisa A. Carey, Charles M. Perou, Elaine R. Mardis

The S3 Fig legend incorrectly labels Patient A1 as Patient A7. The corrected legend can be viewed below.

## Supporting Information

S3 Fig DNA alterations of matched primary and metastases of patient A1. (A–F): Circos plot displays mutations, copy number, and structural rearrangements in the (A) primary, (B) spinal, (C) lung, (D) liver, and (E) adrenal metastases. Translocations with significant read coverage include shared (green) and private (red) interchromosomal and shared (purple) and private (blue) intrachromosomal translocations.
(PDF)
