# Supplementary material for: Tumor Evolution in Two Patients with Basal-like Breast Cancer: A Retrospective Genomics Study of Multiple Metastases
Source: PLoS Med. 2016 Dec 6;13(12):e1002174. doi: 10.1371/journal.pmed.1002174 (PMC5140046; doi:10.1371/journal.pmed.1002174)
Supplement: S1 Appendix — In-depth description of the capture array design, paired-end library preparation, and solid phase capture. Further description of bioinformatic methods for clonality and determining significantly mutated genes. (DOCX) [file pmed.1002174.s001.docx]

Supplementary Materials and Methods

Capture array design. For small insertions and SNVs, the targeted regions were exactly 200 bp centered on the variant. For small deletions, the deleted sequence plus 100 bp of sequence flanking each end of the deletion were selected. For putative somatic SVs, we requested probes tiled across the predicted breakpoints with 100 bp flanking sequence on either side of these locations. For larger insertions a single region was requested, but for translocations, deletions, and inversions, we requested two targets, one for the breakpoint on each side of the events. Roche NimbleGen design parameters allowed for probes with up to five additional sequence matches elsewhere in the genome.

Paired-end library preparation for solid-phase capture validation. Genomic DNA (50 ng) was sent to QIAGEN for whole genome amplification (QIAGEN, Germantown, MD). Whole genome amplified (WGA) DNA samples (3 μg) were suspended in 1X MicroTube (6x16mm), AFA Fiber with Snap-Cap tubes (CovarisTM) using the Covaris S2 DNA Sonicator (Covaris, Inc. Woburn, MA). Fragmentation conditions were in 50 μl reaction volumes and performed at 4^o^C. The S2 parameters were set to the frequency sweeping mode using two successive, 60 second, acoustic treatments of: 1) duty cycle=20%, intensity=5, cycle/bursts=500. The DNA fragments were end repaired by directly adding 2 μl of the DNATerminator® End Repair Enzyme cocktail (Lucigen Corp. Madison, WI) to the MicroTube and incubating at room temperature for 30 minutes. The end-repair reactions were purified using Solid Phase Reversible Immobilization (SPRI) by adding 90 μL (1.8X sample volume) of AMPureXP beads (Agencourt Bioscience, Beverly, MA) to the end-repair reaction. The bead milieu was incubated for 5 minutes at room temperature, allowing the DNA to bind to the beads. The beads were then immobilized against a magnetic particle collector (MPC; Invitrogen, Carlsbad, CA), washed twice with 500 μl of 70% ethanol, and air dried for 5 minutes. DNA fragments were released by adding 32 μl of 10 mM Tris-HCl (pH 8.0).

End-repaired DNA fragments were tailed with 0.2 mM deoxyadenosine triphosphate in the presence of 15U Klenow exo- (New England Biolabs, Worcester, MA) at 37^o^C for 30 minutes. Each 50 μL reaction was mixed with 90 μl AMPureXP beads, as described above (Agencourt Bioscience, Beverly, MA). DNA was eluted in 20 μL of 10 mM Tris-HCl (pH 8.0). Illumina adapters were ligated to A-tailed DNA fragments per the manufacturers protocol (Illumina Inc, San Diego, CA) in the presence of 1X Quick Ligase Buffer and 10,000U Quick Ligase (New England Biolabs, Worcester, MA) at 25^o^C for 15 minutes. Small fragments less than 100 bp and unligated adapters were removed from the mix by AMPureXP bead purification (as reported above). The adapter-ligated DNA samples were eluted in 20 μL of 10 mM Tris-HCl (pH 8.0).

Illumina library amplification. A PCR amplification master mix was prepared using 160 nM of the Illumina paired-end oligonucleotides 1.0 in 1X PhusionTM High Fidelity PCR Master Mix with HF (PE1.0: 5’-AATGATACGGCGACCACCGAGATCTACACTCTTTCCCTACACGACGCTCTTCCGATCT)and 2.0(PE2.0:5’-CAAGCAGAAGACGGCATACGAGATCGGTCTCGGCATTCCTGCTGAACCGCTCTTCCGATCT) in 1X Phusion™ High Fidelity PCR Master Mix with HF Buffer (New England Biolabs, Beverly, MA). Four reactions per sample were prepared using 5 μl of adapter- ligated DNA in 45 μl of PCR amplification master mix and PCR amplified with an initial thermal denaturing step of 98 ^o^C for 30 seconds followed by 5 rounds of amplification: 98 ̊C, 15 seconds, 60^o^C, 30 seconds, and 72^o^C, 30 seconds. After PCR amplification, we enriched for DNA fragments in the 300-500 bp range using two SPRI isolation procedures. Firstly, to remove DNA fragments > 500 bp, we added 0.6X volumes (30 μl) of AmpureXP beads and incubated for 5 minutes at room temperature allowing the DNA to bind to the beads. The beads were then immobilized, and the PCR supernatant was aspirated and transferred to the second sizing solution, which was used to remove DNA fragments less than 300 bp.

We aliquoted 60 μL AmpureXP beads into a new 1.7 ml microfuge tube, incubated the bead solutions on the MPC for 3 minutes, aspirated and discarded the supernatant. To the bound beads, we mixed an additional 0.2X volume of AmpureXP beads to the existing beads on the MPC, creating the second sizing solution.

After incubating the PCR supernatant with second sizing solution for 5 minutes, the SPRI beads were bound using the MPC, the supernatant aspirated and discarded leaving magnetic particles coated with 300-500 bp DNA fragments. The beads were washed twice with 500 μL of 70% ethanol, allowed to air dry and eluted in 20 μL of Nuclease-Free Water (Ambion, Austin, TX). The sample was then quantified on a NanoDrop Spectrophotometer (NanoDrop Products, Wilmington, DE).

Solid phase capture. 300 μl (1μg/μl) of Cot-1 DNA (Invitrogen, Carlsbad, CA) was mixed mplified Illumina library and completely desiccated in a SpeedVac set on 60°C for 1 hour. The sample was then hydrated with 9.2 μl of Nuclease-Free Water (Ambion, Austin, TX) and incubated at 70°C for 10 minutes. Following incubation, 18.5 μl of 2X Hybridization Buffer, 7.3 μl of Hybridization Component A (Nimblegen, Madison, WI), and 1 μl of 1mM PE1.0 and 1mM PE2.0 were added to the sample. The mixture was then denatured at 95°C for 10 minutes and incubated at 42°C until ready for loading.

The NimbleGen HD2 array (NimbleGen, Madison, WI), was prepared by placing the array face-up in the Precision Mixer Alignment Tool (PMAT) (NimbleGen, Madison, WI). A NimbleGen HX1 mixer was snapped into place on the top of the PMAT with the adhesive gasket removed. The PMAT was closed, concomitantly aligning the HX1 mixer onto the HD2 array. The array was placed into a NimbleGen Hybridization System 4 (NimbleGen, Madison, WI), and using a Microman 100 Pipette with CP100 tips (Gilson, Middleton, WI), 37 μl of hybridization solution was loaded into the fill port. After loading, exposed sample at the fill and vent ports was removed with a clean tissue. The two ports were then closed with port seals (NimbleGen, Madison, WI). The hybridization bay clamp was then closed, the Mixing Panel was set on mix mode B, and library was hybridized to the array for 72 hours at 42°C

After hybridization, the array-mixer was disassembled in a reservoir containing 100 ml of pre-eluent washes to minimize non-specific carry-over. These washes included: 1) 32 ml of NimbleGen Wash Buffer 2 (47°C) followed by subsequent washes to minimize non-specific carry-over. These washes included: 1) 32 ml of Nimblegen Wash Buffer 2 pre-heated to 47°C, inverting the wash tube 10 times at a rate of 1 inversion per second, 2) Two consecutive washes with 32 ml of NimbleGen Stringent Wash Buffer pre- heated to 47°C, inverting 10 times at a rate of 1 inversion per second and incubating at 47°C for 5 minutes 3) 32 ml of Nimblegen Wash Buffer 1 at room temperature, inverting for 2 minutes at a rate of 1 inversion per second, 4) 32 ml of NimbleGen Wash Buffer 2 at room temperature, inverting for 1 minute at a rate of 1 inversion per second, and 5) 32 ml of NimbleGen Wash Buffer 3 at room temperature, inverting the wash tube 10 times at a rate of 1 inversion per second.

The array was then placed in an Elution System (NimbleGen, Madison, WI), and covered with the EL1 Elution Chamber (NimbleGen, Madison, WI). Captured library fragments were released from the probe array by adding 900 μl of 0.125 N sodium hydroxide and incubating for 10 minutes at room temperature. The eluant was pipetted from the EL1 Elution Chamber, neutralized by splitting the material into two tubes (~450 μl) containing 16 μl of 20% acetic acid in 500 μl of Buffer PB (QIAGEN, Germantown, MD), and finally purified using MinElute columns (QIAGEN, Germantown,MD), Captured DNA was recovered from MinElute columns in 20 μl of 10 mM Tris-HCl (pH 8.0).

The captured fragments were amplified in the PCR consisting of 25 μl 2X Phusion HF Master Mix (Finnzymes, Woburn, MA), 20 μl captured DNA, 1 μl each 8 μM PE1.0 and PE2.0, and 3μl of water. Amplification used the following parameters: initial denaturation, 98°C for 30 seconds followed by 16 cycles of denaturation at 98°C for 10 seconds, primer annealing at 60°C for 30 seconds, and extension at 72°C for 30 seconds. After a final elongation at 72°C for 2 minutes, the reactions were pooled and purified with 1.8X volume (360 μl) of AMPure XP beads, washed twice with 500 μl 70% ethanol, and air dried for 5 minutes. The capture libraries were released from the beads with 20 μl of 10 mM Tris-HCl (pH 8.0).

Illumina library quantification was completed using the KAPA SYBR FAST qPCR Kit (KAPA Biosystems, Woburn, MA). The qPCR consisted of 12 μl of KAPA SYBR FAST Master mix (KAPA Biosystems, Woburn, MA), 4 μl of Nuclease-Free Water (Ambion, Austin, TX), and 4 μl of captured library diluted 1:1000 with 10 mM Tris-HCl (pH 8.0). The qPCR result was used to determine the quantity of library necessary to produce ~180,000 Clones on a single lane of the Illumina GAIIx. One lane of 100bp Paired-End data was generated for each captured sample.

Establishing clones and subclones per tumor sample

For estimating the division of clones and subclones within each tumor sample, we use a kernel density analysis applied to the variant allele frequencies (VAFs) of somatic SNVs in copy-number neutral regions. We predicate that accurate VAFs of specific mutations give an indication of the history of that mutation within the population of tumor cells. In other words, mutations occurring earlier in the development of a tumor will be present in a larger percentage of the tumor’s cells, and thus will exhibit larger numbers of variant reads (higher VAF) in the sequencing data than the number of variant reads observed from more recent mutations (lower VAF). By observing Clones of plotted VAFs for a particular sample, we are able to infer the clonal structure of that tumor.

In order to obtain accurate VAFs for the kernel density analysis, we use only the VAFs of SNV sites with deep coverage (>100x) in the capture validation data. Efforts were also made to exclude somatic SNVs from regions containing copy number alterations identified in WGS data, which would disrupt the accuracy of VAF measurements and would be hard to predict exactly. This exclusion was performed by running Varscan 2 on the whole-genome sequencing data, and then for each chromosome, plotting the VAFs from both the tumor and normal samples at sites where the normal sample’s VAF fell between 40% and 60%. Sites in regions exhitibing loss of heterozygosity were also excluded. The remaining SNVs were further segregated according to their segmented copy number states as predicted by cnvHMM (states of copy number equal to 1, 2, 3, or 4), and each copy number state was analyzed individually.

For each copy number state in the tumor, a kernel density estimate (KDE) plot was drawn for tumor VAFs using the density function in R. A customized R function evaluated each KDE plot to determine the number of significant peaks in the KDE representing the copy number neutral, or diploid regions. These peaks indicate Clones of similarly-aged mutations, and thus served as an estimation of the number and relative composition of clones and subclones present in each tumor. We also used a computational tool SciClone (<https://github.com/genome/>sciClone) to infer the numbers of subclones in each tumor and assigned clonal fractions (“Clones”) to each of the metastatic sample.

Determining significantly mutated genes.

The SMG test identifies genes that have significantly higher somatic and germline mutation rates than background. For the purposes of our algorithm, variant counts were divided into seven categories: A and T transitions, A and T transversions, CpG transitions, CpG transversions, C and G (non CpG) transitions, C and G (non CpG) transversions, and lastly, an “indel” category, for which we used the entirety of the covered space in the sample-set when comparing indel-affected bases versus available bases. To determine the BMR for each of the mutation categories, we divided the mutation number found in each category by the total number of covered bases available across the cohort in which such a call can be made. For each gene, p-values were calculated for each mutation category by assessing the significance of the MR versus the BMR for that category. Three statistical methods were then used to summarize the per-category p-values, (a convolution test, a Fisher’s combined P-value test, and a likelihood ratio test). In addition, false discovery rates (FDRs) were calculated for each combined p-value. We evaluated our SMG test results by establishing a p-value or FDR threshold, and filtering the results based on these thresholds. To account for hypermutated samples, the ability to sub-group cases based upon similarities in their overall mutation counts was utilized, and P-values were calculated for mutated genes in each sub-group independently. All P-values were combined using the same methods as described in Dees *et al*.

Phylip

There are numerous well-established tools for population-based phylogenetic analysis and these can be re-purposed for cancer phylogenetics [2]. We selected the "Phylogeny Inference Package", aka PHYLIP [3], because it is now a somewhat common system for cancer phylogenetic analysis [4,5]. We examined PHYLIP's various tools for analyzing the breast cancer data sets, but only its maximum likelihood method, "contml", is implemented such that allele frequency information is used. Allele tallies observed in the digital sequencing data furnish approximations for true allele frequencies and this represents significantly more information than alleles alone. That is, as a real-valued variable, an allele count gives vastly better resolution over an essentially binary indicator variable (reference or variant). Consequently, we used "contml" for all analyses reported here.

In order to further assess the legitimacy of this strategy, we first undertook a set of preliminary, nucleotide-only analyses for the data using all 3 approaches in order to evaluate any level of dependence of the results upon the actual calculation method. In all cases, the phylogenetic topologies did not vary with approach. Moreover, none of the various well-known pitfalls of phylogenetic analysis, e.g. "long branch attraction" in parsimony [6], seemed to be present for these data. Consequently, the choice of which analysis method to use did not seem to depend heavily upon their underlying technical aspects. Because of information content discussed above, we subsequently concluded that "contml" was the most preferable. Indeed, we note the importance of allele frequency information, as the topologies for the maximum likelihood method were fundamentally different from their corresponding allele-only counterparts.

Nucleotide sites are considered to be independent of one another by "contml", meaning the overriding factor for the calculation is consistency of the inputs. Procedurally, frequencies are simply concatenated in order of position within the genome and subsequently written as input files in PHYLIP format. The normal sample is simply the concatenation of reference bases at each site where any sample in the collection manifested a variant. Each tumor sample sequence is the correspondingly ordered string of its own variant and reference bases.

PHYLIP tools output tree topologies in a graph-theoretical ASCII notation known as "Newick format". While the PHYLIP package provides various post-processing tools to convert Newick information into graphical formats for display, the results are somewhat crude. Consequently, we use PHYLIP's facility for writing graphics information out to standardized formats, in particular the "fig" format, and then do further downstream graphical processing with other tools to generate phylogenetic trees.

Because passenger mutations are incidental, they are often presumed to function collectively as a molecular clock with respect to timing of events in cancer evolution [1]. We therefore used the complete complement of mutations (drivers plus passengers, the latter dominating in number) for each calculation. Consequently, numerical values are taken as indicators of genetic distances between a most recent common ancestor (previous branch-point) and either the current branch-point or the terminus of a sequenced genome.
