# Supplementary figures and images for: Tumor Evolution in Two Patients with Basal-like Breast Cancer: A Retrospective Genomics Study of Multiple Metastases
Source: PLoS Med. 2016 Dec 6;13(12):e1002174. doi: 10.1371/journal.pmed.1002174 (PMC5140046; doi:10.1371/journal.pmed.1002174)

Supplemental Figure 1

A.

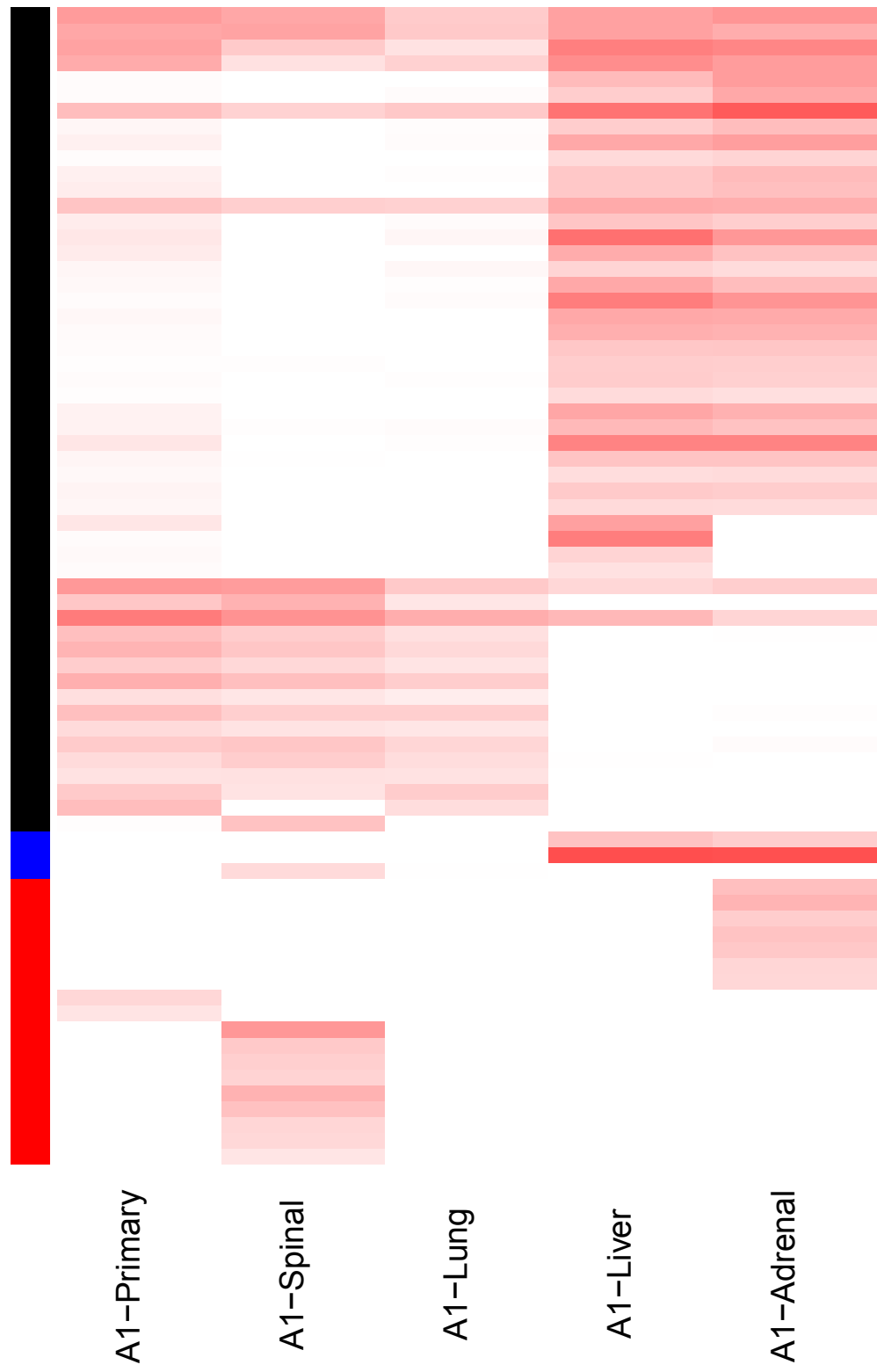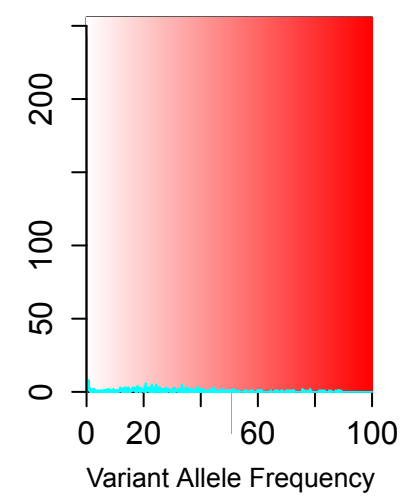

■ Founder Mutation  
■ Metastasis Specific  
■ Private

B.

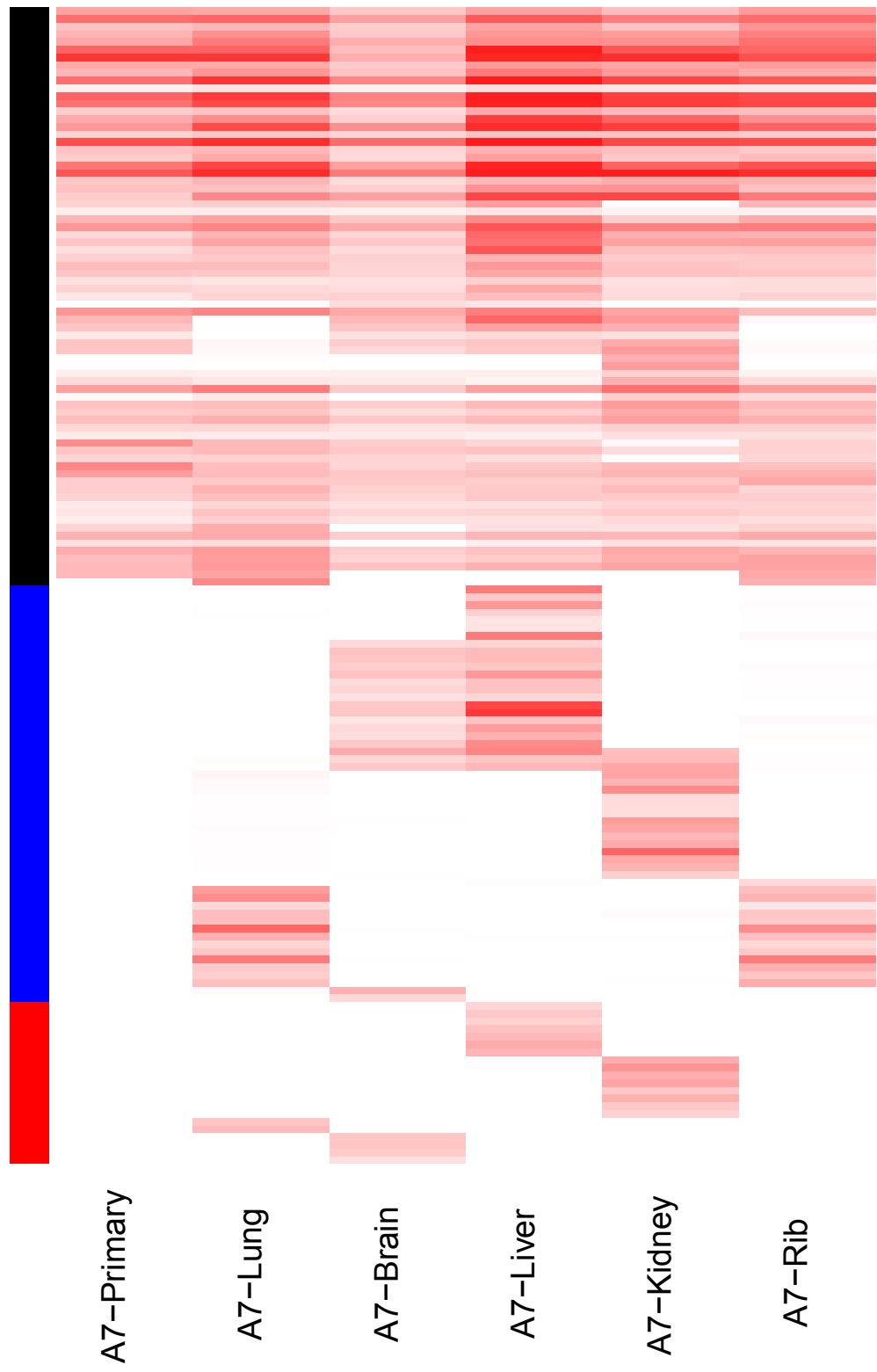

Supplement: S1 Fig — The vertical bar to the left of each heat map designates genes shared with the primary and metastases (black), genes mutated in metastases but not in the primary (blue), and genes private to a single individual metastasis (red) in (A) Patient A1 and (B) Patient A7. (PDF) [file pmed.1002174.s002.pdf]

# Supplemental Figure 2

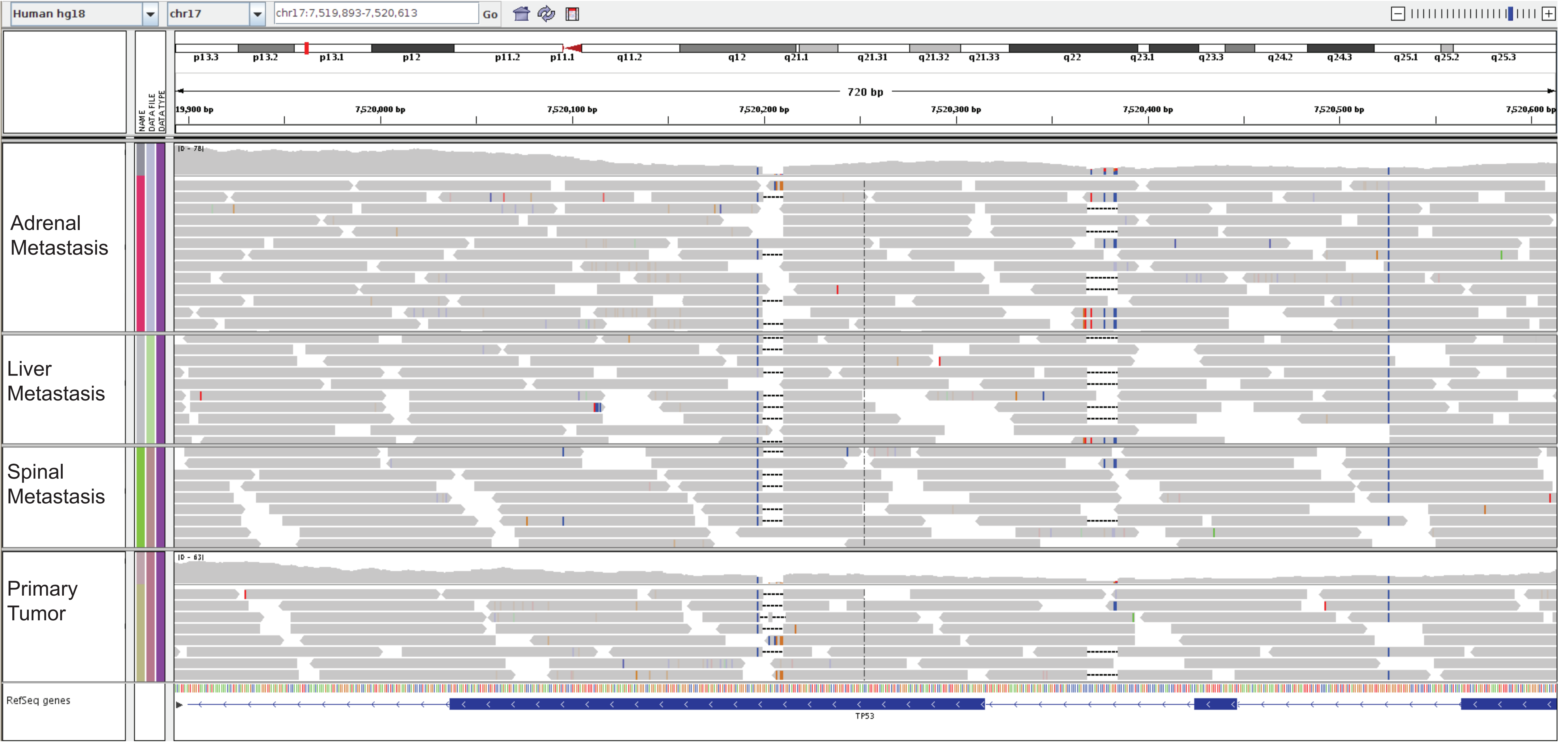

Supplement: S2 Fig — Genome view of the 11 bp deletion of TP53 in Patient A1 at chr17:7,579,474 to chr17:7,579,485, present in the primary tumor and all of the metastases. (PDF) [file pmed.1002174.s003.pdf]

# Supplementary Figure 4

A. A7-Primary

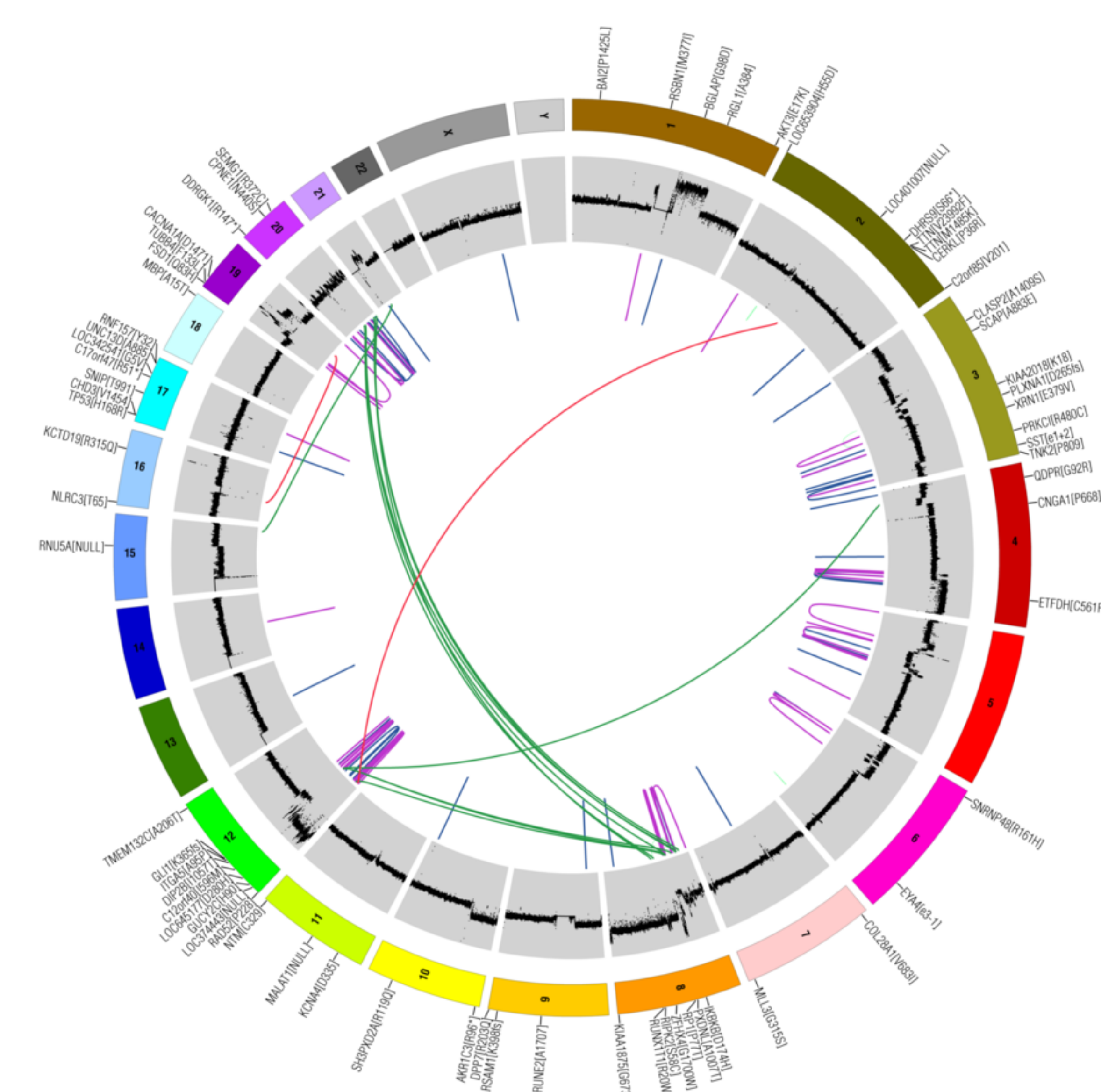

**B.** A7-Rib

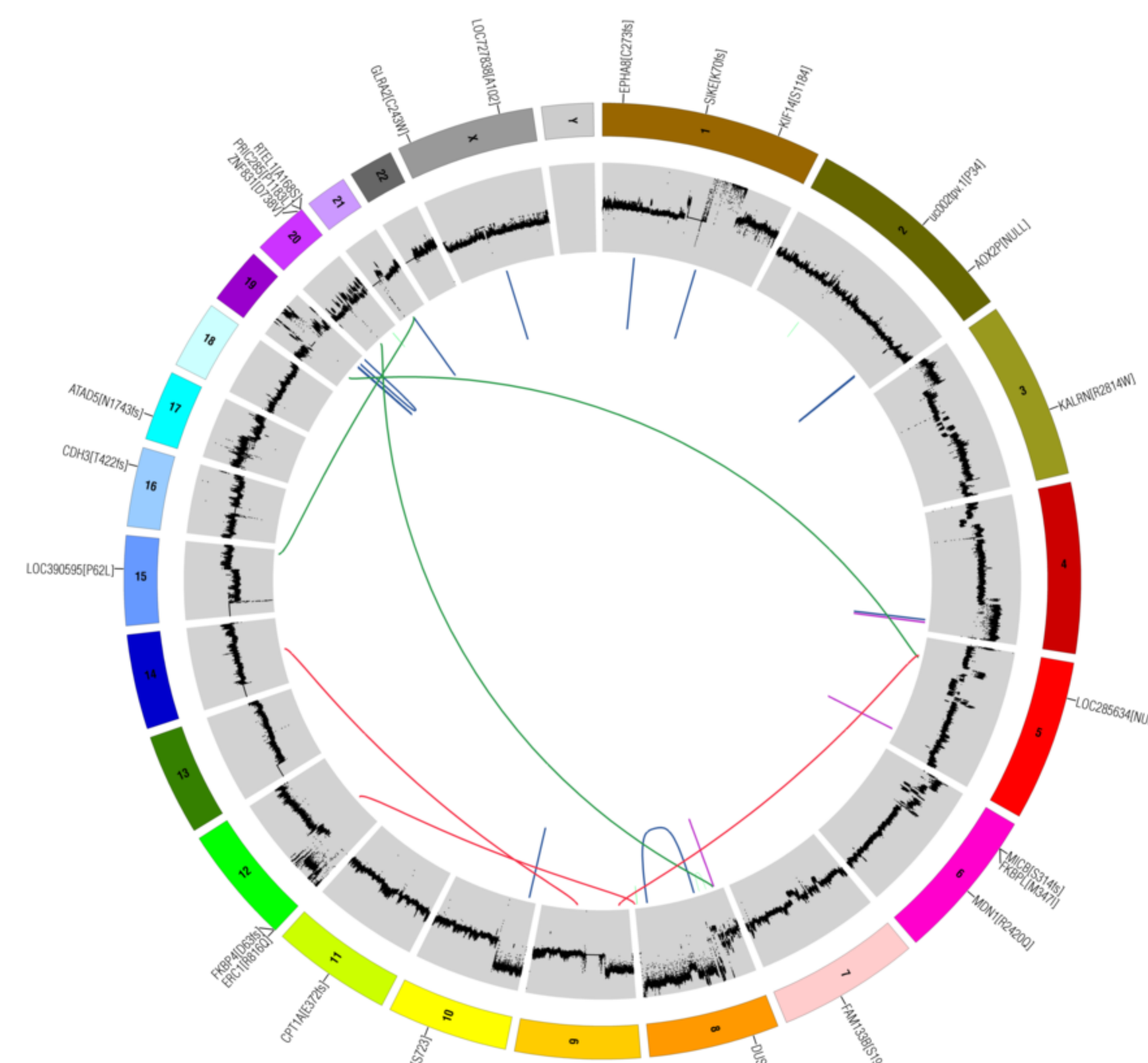

C. A7-Kidney

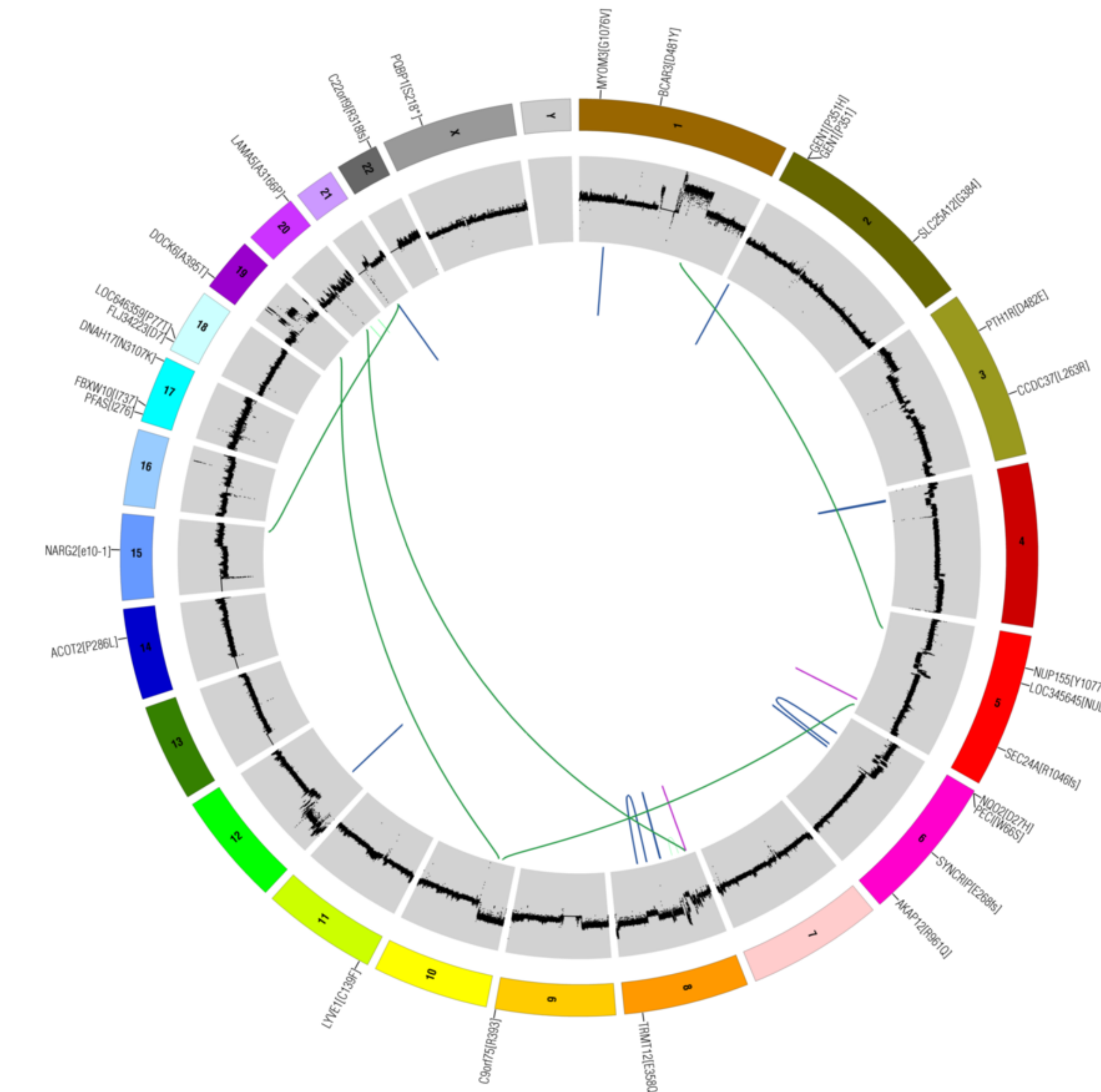D. A7-Liver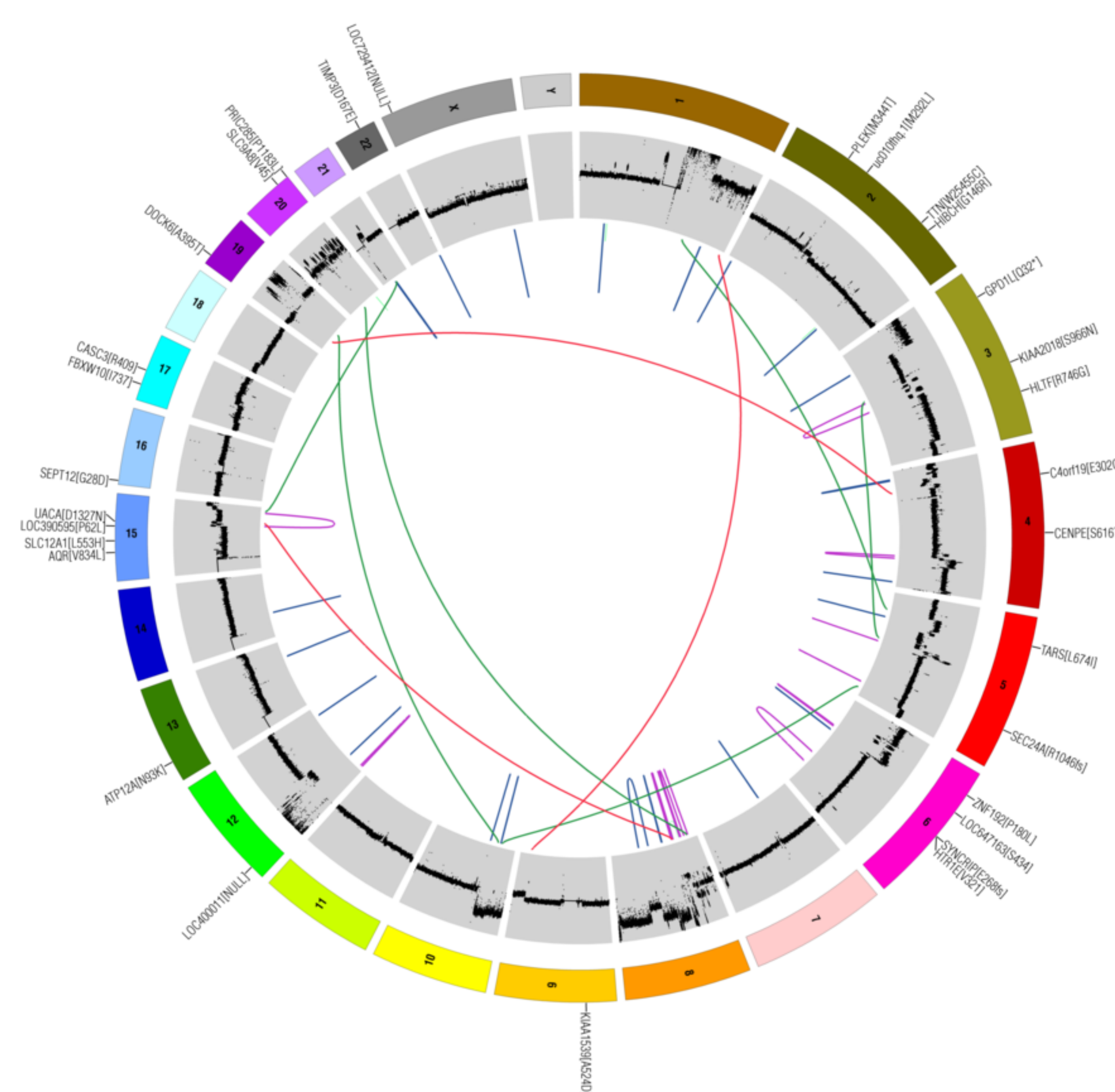

E. A7-Brain

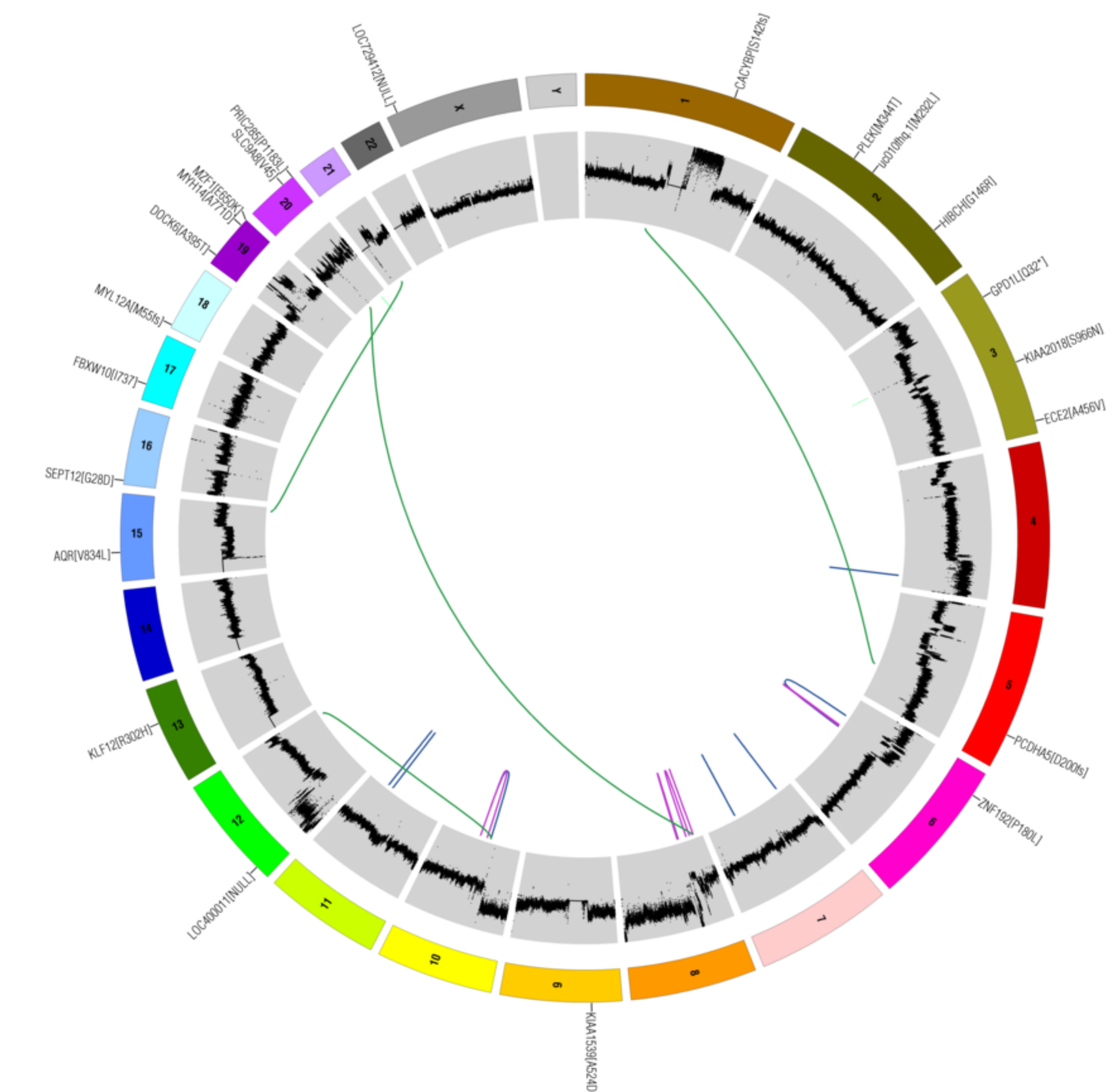

F. A7-Lung

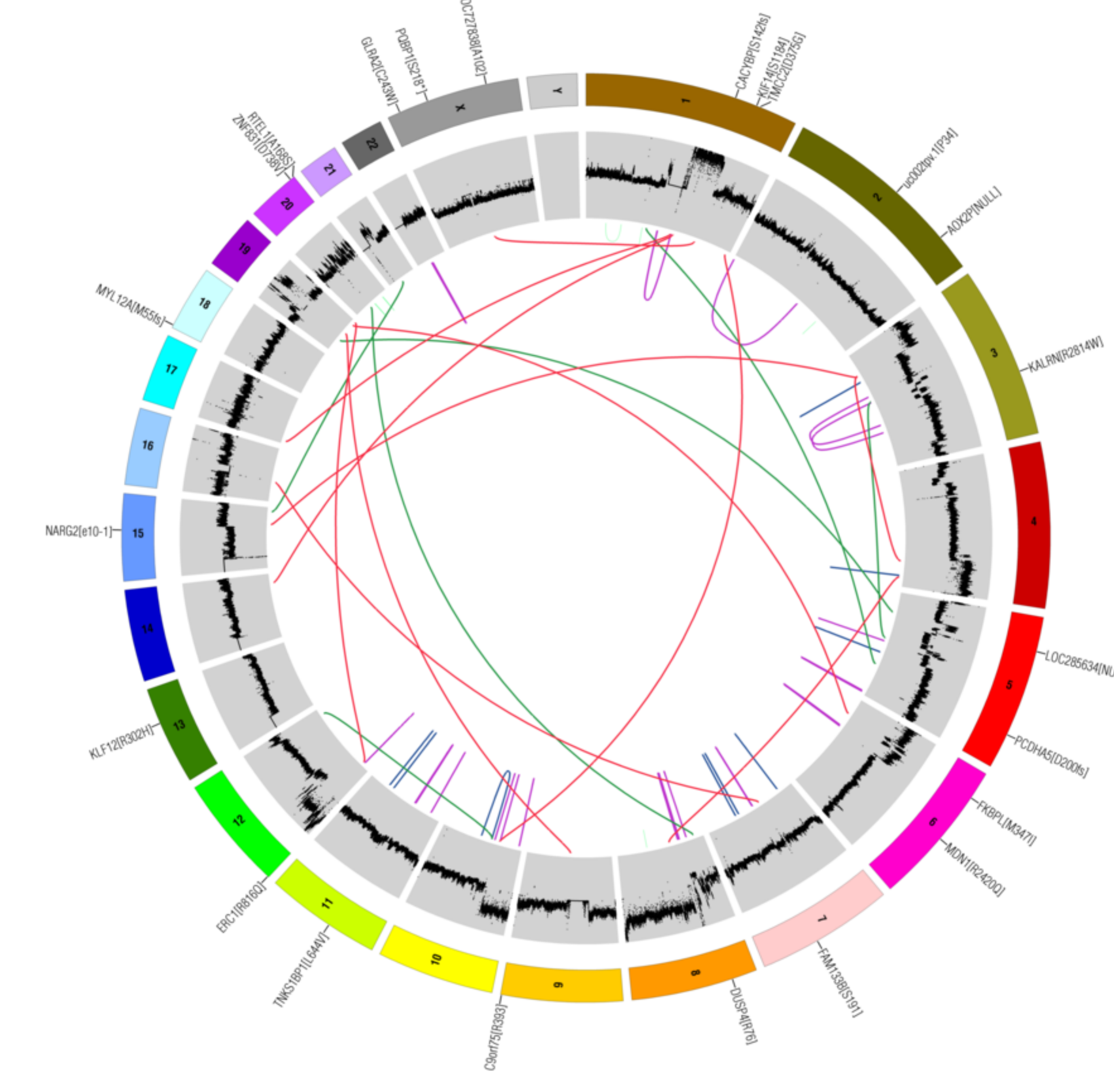

Supplement: S4 Fig — (A–F): Circos plots displaying mutations, copy number landscape, and structural rearrangements (order starting from outside) in the (A) primary, (B) rib, (C) kidney, (D) liver, (E) brain, and (F) lung metastases. Translocations with significant read coverage include shared (green) and private (red) interchromosomal and shared (purple) and private (blue) intrachromosomal translocations. (PDF) [file pmed.1002174.s005.pdf]

Supplemental Figure 5

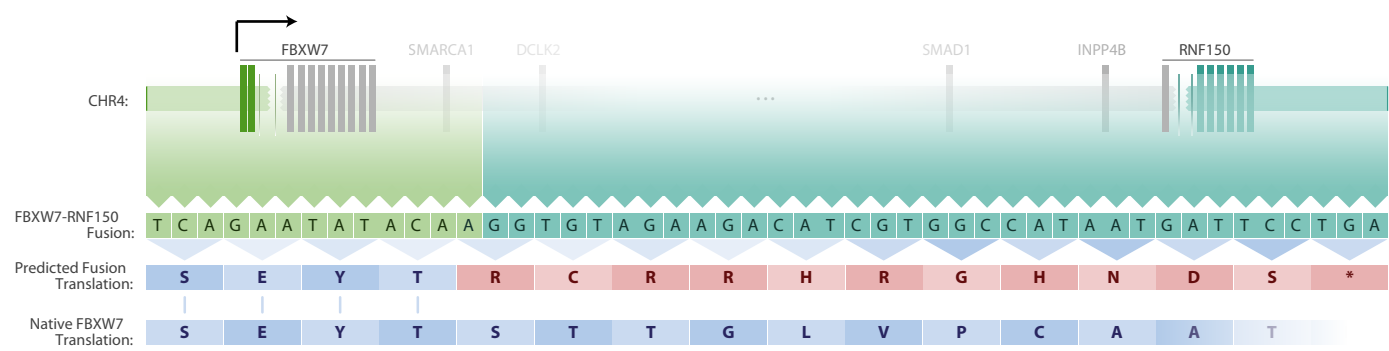

Supplement: S5 Fig — Representative illustration of FBXW7 fusion and INPP4B deletion in all tumors from A7. (PDF) [file pmed.1002174.s006.pdf]

A.

Max (clone cross-sample p) = 0.14126

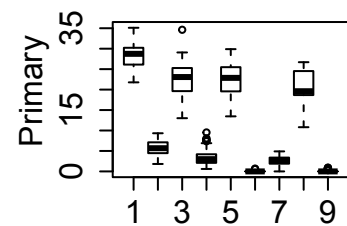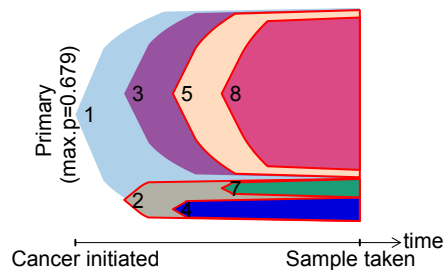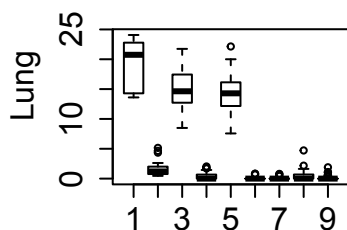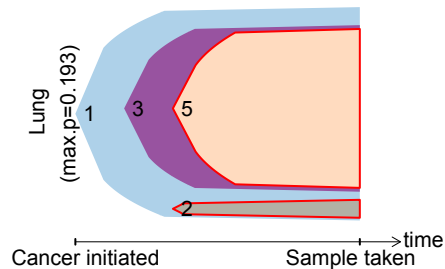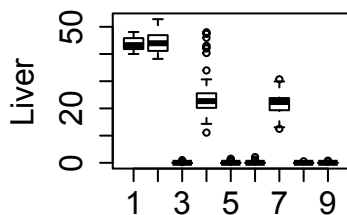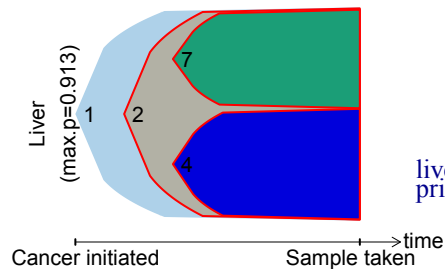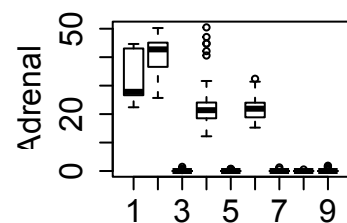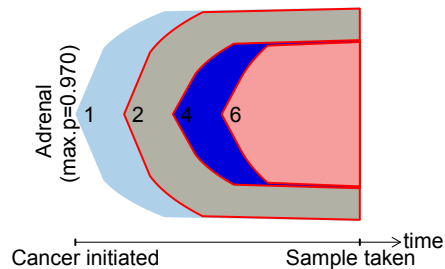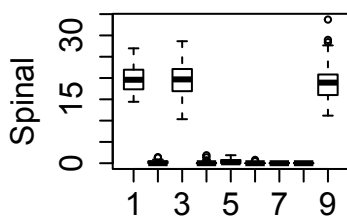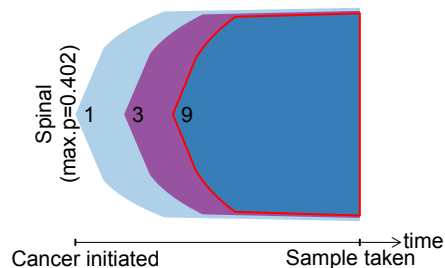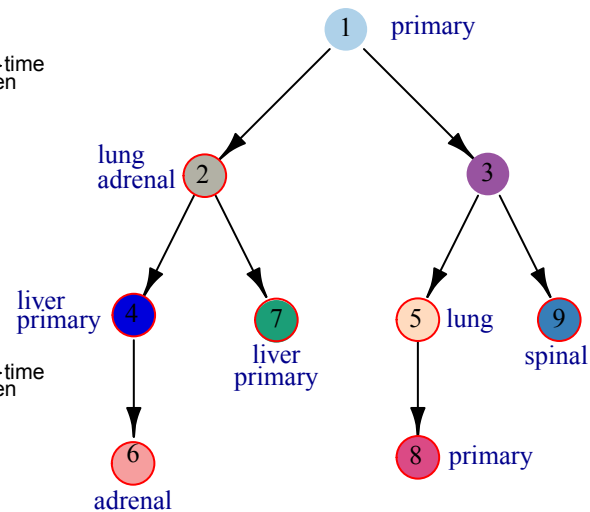

Max (clone cross-sample p) = 0.14176

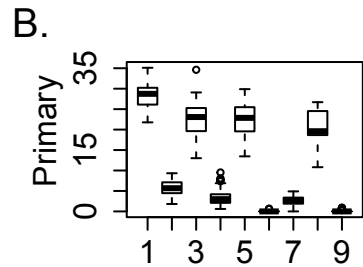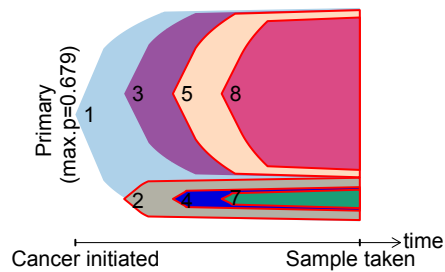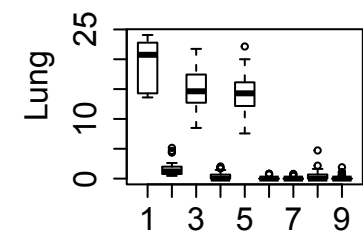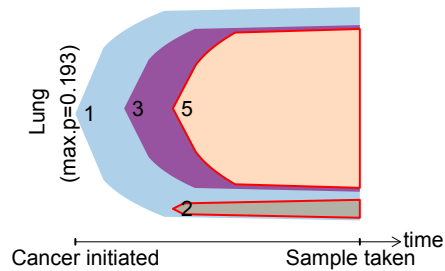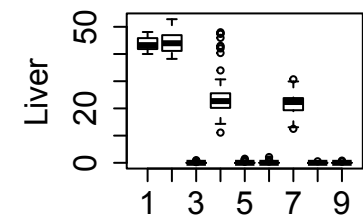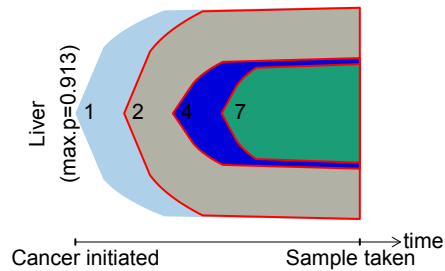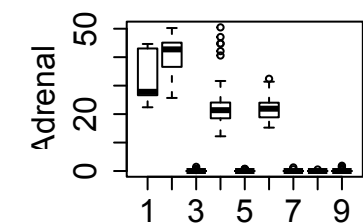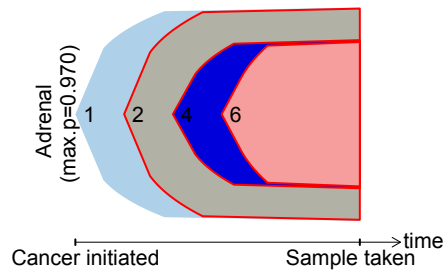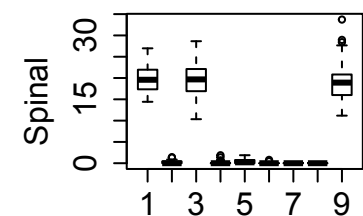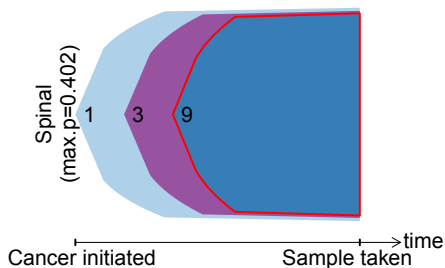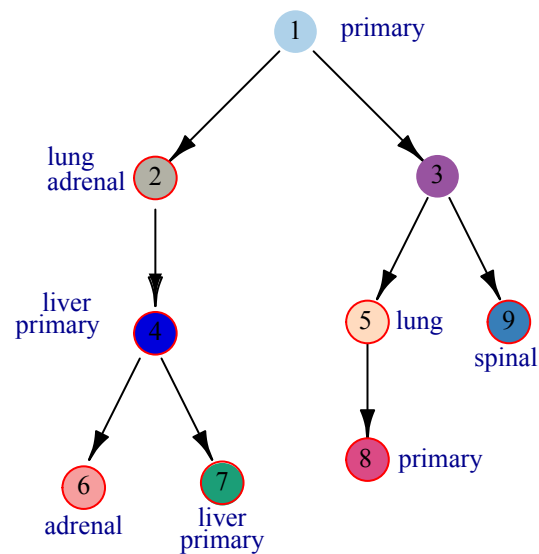

Supplement: S7 Fig — ClonEvol demonstrates that Clones 1 and 2 are founding clones that seed the distant metastases at different percentages. Clone 2 and Clone 3 are exclusive of one another, leading to separate lineages. The proportion of each clone for each tumor is demonstrated by the width of the nested shapes. Two possible models are presented. (PDF) [file pmed.1002174.s008.pdf]

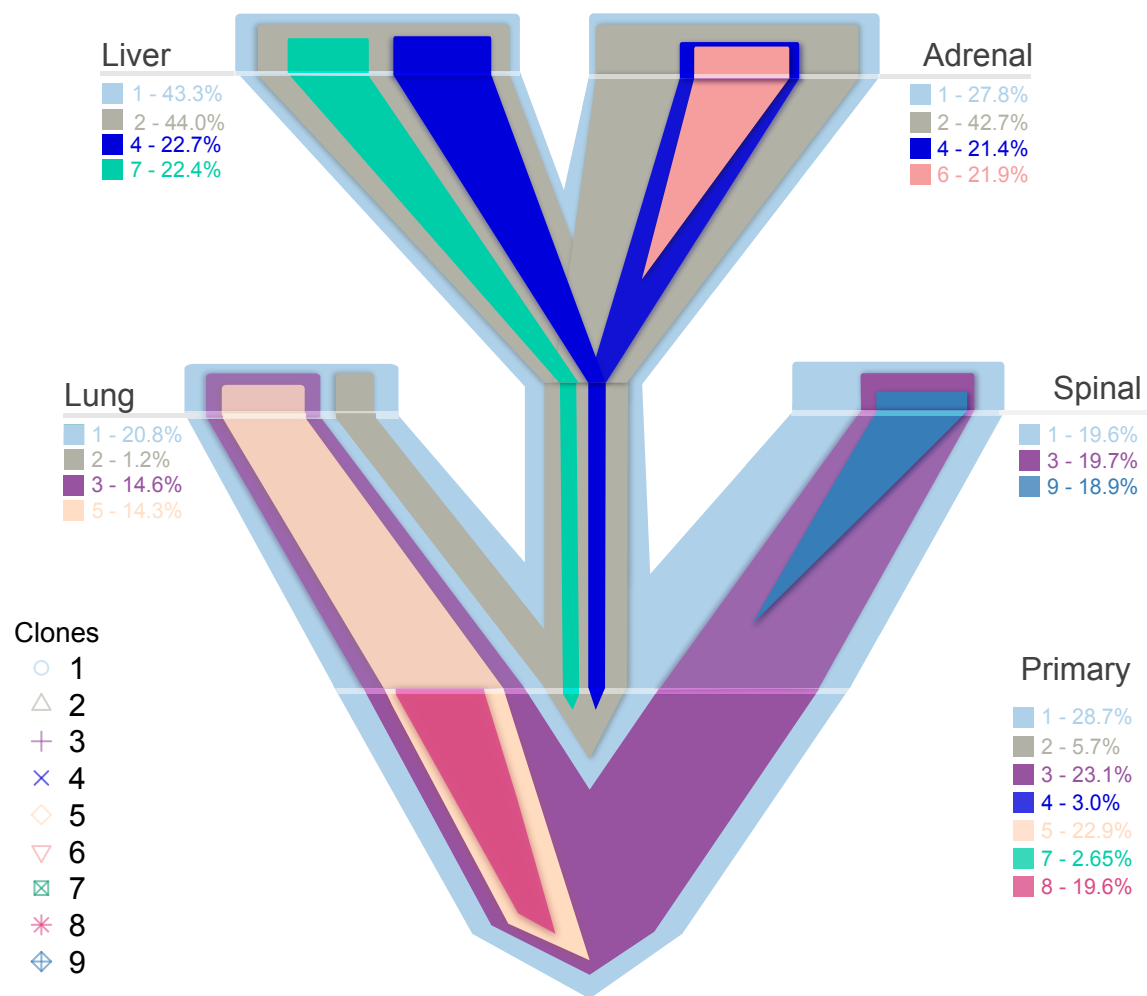

Supplement: S8 Fig — ClonEvol predicted two possible evolutionary lineages of clones in patient A1. The first model is in Fig 5B. The alternative model demonstrating that Clone 7 is independent of Clone 4 is presented. (PDF) [file pmed.1002174.s009.pdf]

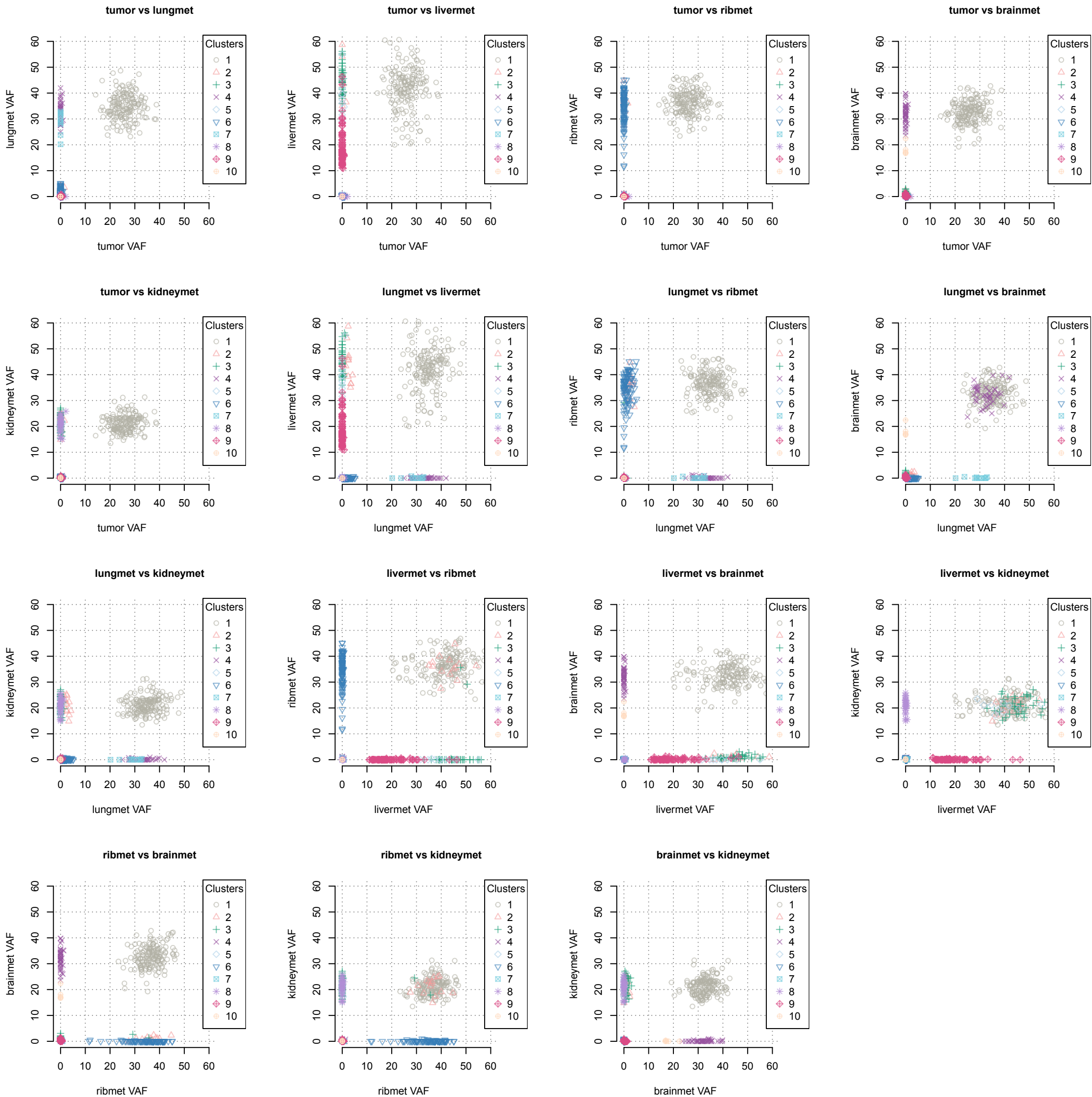

Supplement: S9 Fig — SciClone analysis of only copy number neutral regions demonstrates multiclonal seeding of metastases. The Lung metastasis contains both branches of the clonal tree, predominantly containing Clone 4 but with a small fraction of Clone 2. In Contrast, the rib metastasis contains predominantly Clone 2 with a small minority of Clone 3. Private clones are seen in all metastases. (PDF) [file pmed.1002174.s010.pdf]

Max (clone cross-sample p) = 0.007269

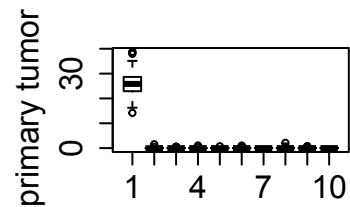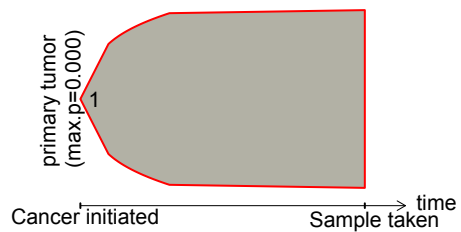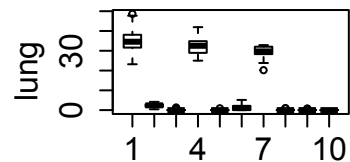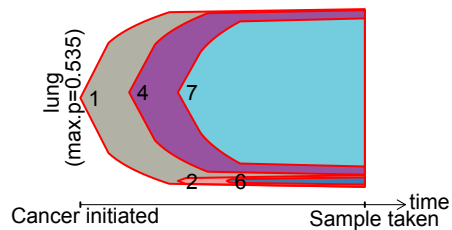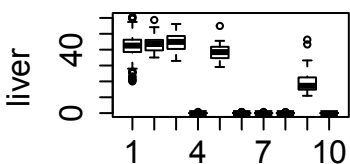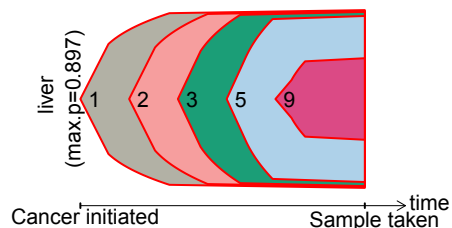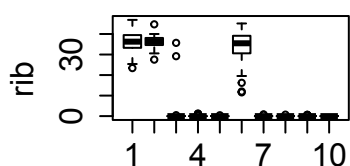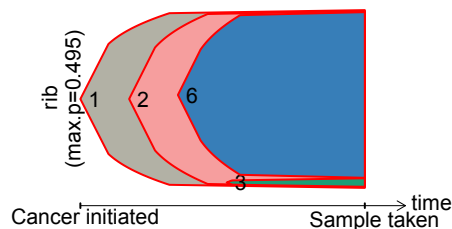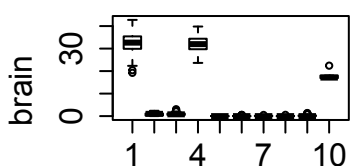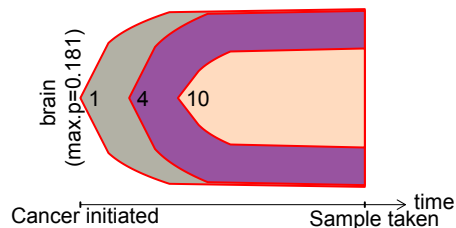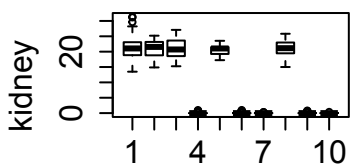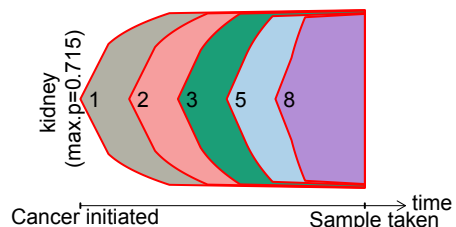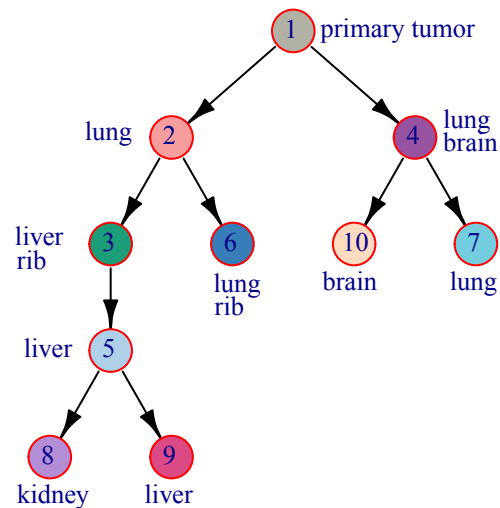

Supplement: S10 Fig — ClonEvol of the copy number neutral mutations from SciClone analysis demonstrates one founding clone leading to a branched pattern of Clones 2 and 4. Private clones are present in all metastases. (PDF) [file pmed.1002174.s011.pdf]
