# Supplementary material for: Tumor Evolution in Two Patients with Basal-like Breast Cancer: A Retrospective Genomics Study of Multiple Metastases
Source: PLoS Med. 2016 Dec 6;13(12):e1002174. doi: 10.1371/journal.pmed.1002174 (PMC5140046; doi:10.1371/journal.pmed.1002174)
Supplement: S6 Fig — SciClone analysis of variant allele frequencies in copy number neutral regions of each tumor using Bayesian beta mixture modeling and multi dimensional clustering of tumors from patient A1. Multiple clones are shared in the primary and metastases, with Clone 1 in the primary and all matched metastases; Clone 2: primary, adrenal, and liver; Clone 3: primary, adrenal, and liver; Clone 4: primary, lung, and spine; Clone 5: primary, adrenal, and liver; Clone 6: primary and lung; Clone 7: adrenal; Clone 8: primary and liver; Clone 9: primary; and Clone 10: spinal. (PDF) [file pmed.1002174.s007.pdf]

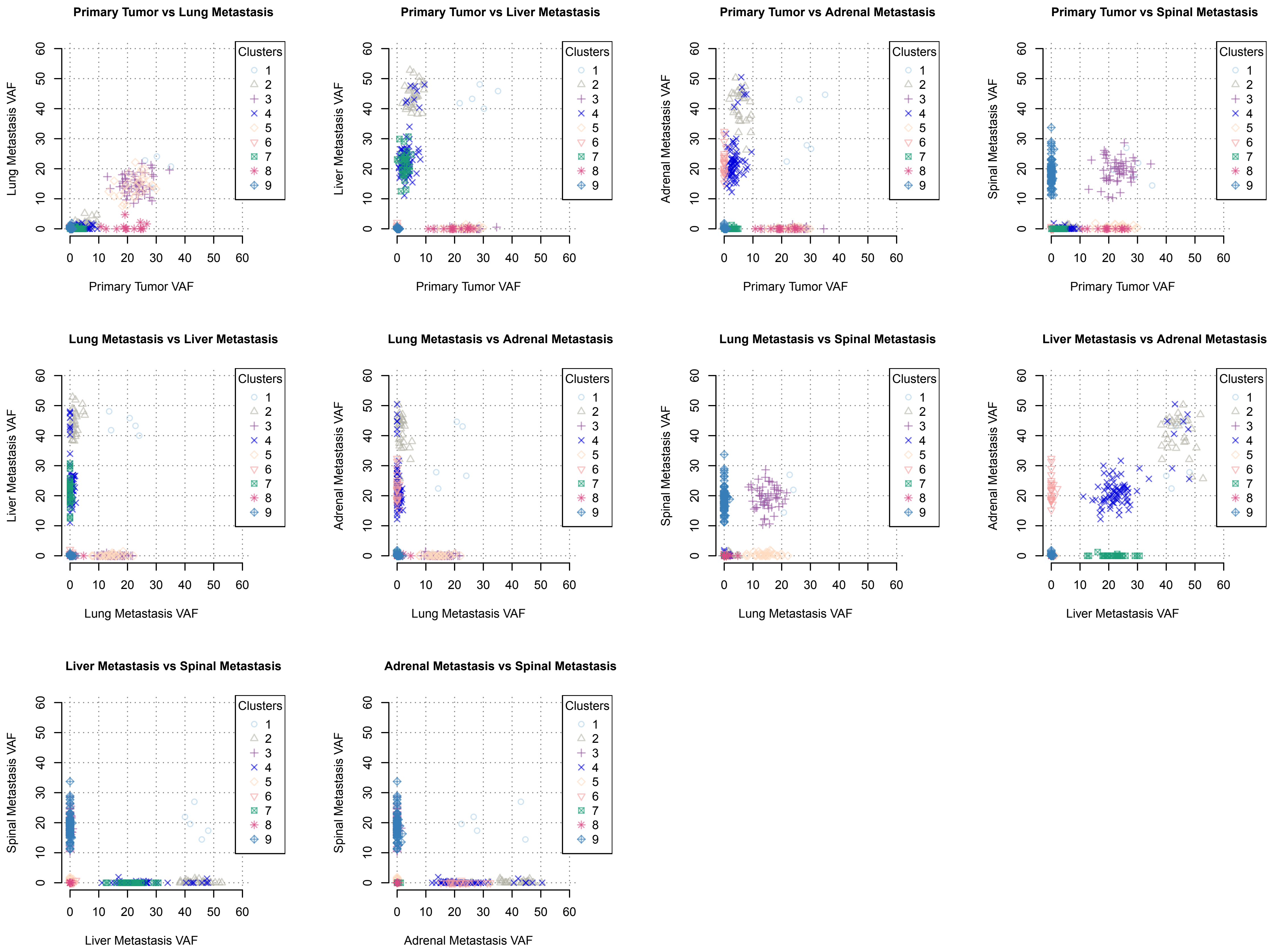
Primary Tumor vs Lung Metastasis
Lung Metastasis VAF
Primary Tumor VAF
Primary Tumor vs Liver Metastasis
Liver Metastasis VAF
Primary Tumor VAF
Primary Tumor vs Adrenal Metastasis
Adrenal Metastasis VAF
Primary Tumor VAF
Primary Tumor vs Spinal Metastasis
Spinal Metastasis VAF
Primary Tumor VAF
Lung Metastasis vs Liver Metastasis
Liver Metastasis VAF
Lung Metastasis VAF
Lung Metastasis vs Adrenal Metastasis
Adrenal Metastasis VAF
Lung Metastasis VAF
Lung Metastasis vs Spinal Metastasis
Spinal Metastasis VAF
Lung Metastasis VAF
Liver Metastasis vs Adrenal Metastasis
Adrenal Metastasis VAF
Liver Metastasis VAF
Liver Metastasis vs Spinal Metastasis
Spinal Metastasis VAF
Liver Metastasis VAF
Adrenal Metastasis vs Spinal Metastasis
Spinal Metastasis VAF
Adrenal Metastasis VAF
Clusters
1
2
3
4
5
6
7
8
9
0
10
20
30
40
50
60
