# Supplementary material for: Tumor Evolution in Two Patients with Basal-like Breast Cancer: A Retrospective Genomics Study of Multiple Metastases
Source: PLoS Med. 2016 Dec 6;13(12):e1002174. doi: 10.1371/journal.pmed.1002174 (PMC5140046; doi:10.1371/journal.pmed.1002174)

Supplemental Figure 3

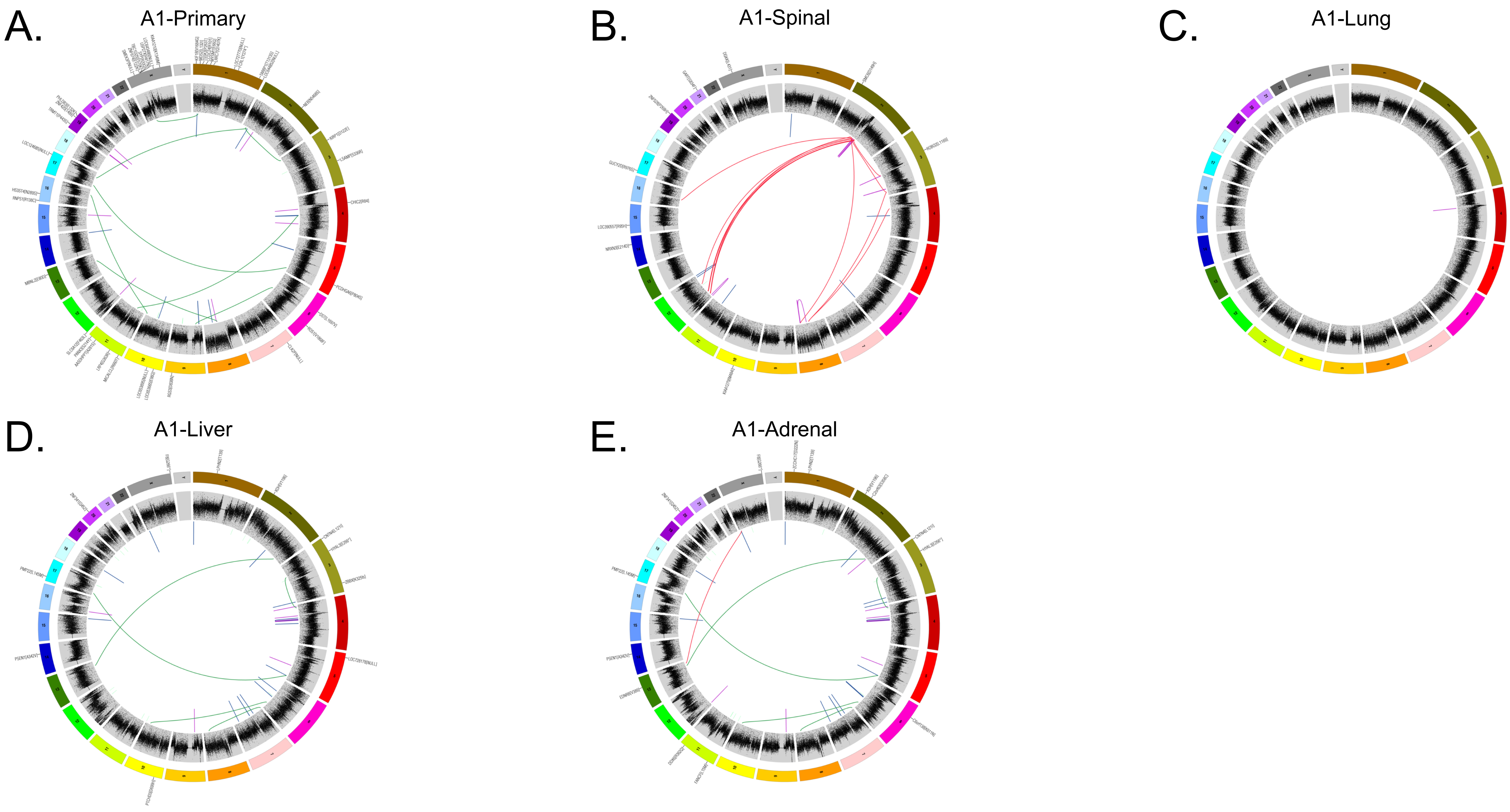

Supplement: S3 Fig — (A–F): Circos plot displays mutations, copy number, and structural rearrangements in the (A) primary, (B) spinal, (C) lung, (D) liver, and (E) adrenal metastases. Translocations with significant read coverage include shared (green) and private (red) interchromosomal and shared (purple) and private (blue) intrachromosomal translocations. (PDF) [file pmed.1002174.s004.pdf]
